# AI-Driven Reconstruction of Online Health Narratives Using Agent-Action-Outcome Modeling: Algorithm Development and Validation

**DOI:** 10.2196/83496

**Published:** 2026-11-13

**Authors:** Ommo Clark, Karuna Pande Joshi

**Affiliations:** 1 Information Systems Department, University of Maryland, Baltimore County, 1000 Hilltop Circle, Baltimore, MD, 21250, United States, 1 410 455 1000

**Keywords:** narrative blindness, health misinformation, natural language processing, narrative analysis, social media, health communication, agent-action-outcome modeling, Labovian theory, Reddit, DistilBERT, computational linguistics

## Abstract

**Background:**

People share health experiences online as stories, not isolated facts. Most computational systems reduce these narratives to keywords or claims, overlooking the agency, causality, and interpretation that determine whether health information is safe or potentially harmful to act on. Content can use medically accurate terminology while describing unsafe behavior, rendering it invisible to fact-checking and keyword approaches. This limitation, which we term *narrative blindness*, constrains the ability of computational systems to extract, represent, and reason over narratives as coherent wholes.

**Objective:**

This study introduces the Narrative Builder, a computational framework that reconstructs unstructured online health stories into machine-readable agent-action-outcome (AAO) narrative graphs, preserving structural, causal, and interpretive dimensions. We formalize narrative blindness as a measurable property of natural language processing systems and evaluate whether theory-grounded reconstruction can address it.

**Methods:**

We operationalized Labov and Waletzky’s sociolinguistic narrative theory within a computational AAO schema, enriched with interpretive dimensions from psychological and clinical communication frameworks. From 5386 Reddit health threads (spanning February 2019 to November 2024), 2 domain experts (a biomedical researcher and a physician) annotated a gold-standard dataset of 2000 segments from 704 threads, with interrater reliability (Krippendorff α) of 0.75 to 0.82 across all annotated tasks (0.78-0.81 for the 4 interpretive classification tasks). A dual-model architecture combining BioBERT (Bidirectional Encoder Representations From Transformers for Biomedical Text Mining) with a conditional random field (CRF) layer for AAO extraction and multitask DistilBERT for interpretive classification was evaluated using 5-fold grouped cross-validation, grouped on the annotation-level key to prevent data leakage, with a separate held-out test set and a McNemar test against term frequency–inverse document frequency (TF-IDF) and BioBERT-without-CRF baselines.

**Results:**

The Narrative Builder achieved a span-level *F*_1_-score of 0.850 for AAO extraction on the held-out test set, with per-type *F*_1_ of 0.90 (action), 0.83 (agent), and 0.77 (outcome), outperforming the TF-IDF baseline (0.781; *P*<.001). Labovian stage classification reached macro-*F*_1_ of 0.501, and interpretive dimensions reached macro-*F*_1_ values of 0.466 (emotion), 0.528 (stance), and 0.465 (tone). Clinical-value extraction achieved a precision of 0.90, and Unified Medical Language System concept normalization reached 100% coverage across 12,101 queries. A rule-based causal direction classifier identified 17.3% of segments as candidate outcome-to-action (inverted) patterns, though validation showed limited precision (0.277), and this distribution is reported as exploratory. Single-rater expert review of generated graphs indicated narrative coherence (mean 0.89, SD 0.08) and medical plausibility (mean 0.91, SD 0.07).

**Conclusions:**

The Narrative Builder reads health stories as coherent wholes rather than isolated facts, producing AAO narrative graphs that retain the agents, actions, outcomes, and emotional framing that determine how individuals interpret and act on health information. By formalizing narrative blindness as a measurable property and reconstructing narrative structure with strong extraction performance, this work establishes narrative reconstruction as a feasible complement to fact-based health informatics, providing structured input for downstream credibility assessment.

## Introduction

### Background

People rarely describe their health experiences online as isolated facts; they tell stories. Consider a Reddit post: “I just turned 27 and they told me my blood pressure is dangerously high. I don’t understand how this is happening to me.” In this brief account, the narrative conveys a medical finding (hypertension), an emotional response (fear), an implicit expectation violation (too young for chronic disease), and an unspoken plea for guidance. Traditional fact-checkers might verify “high blood pressure exists” yet remain blind to the fear, youth confronting chronic disease, and contextual meaning shaping interpretation and action. This gap between what computational systems extract and what humans understand represents a growing challenge for digital health.

The digital transformation of health information has changed how people navigate illness, with search engines and social media functioning as de facto triage tools [1-3]. On Reddit, which hosts over 444 million weekly active users and 116 million daily unique visitors as of late 2025, health information manifests predominantly as personal stories interweaving symptoms, actions, and outcomes through lived experience [4-6]. These stories leverage “narrative transportation,” making them more memorable and persuasive than statistical presentations [7-9]. Our prior survey reveals that while users acknowledge misinformation concerns, peer narrative relatability often supersedes institutional source reliability [10,11]. Users match symptoms to stories, adopt diagnoses from the narrator, and make treatment decisions based on anecdotes, with no indication that this behavior will cease. Thus, the goal cannot be preventing this behavior but ensuring the information encountered is credible and trustworthy.

While health misinformation is often evaluated at the level of isolated factual claims, much of the health information shared online takes the form of personal stories. We define *narrative blindness* as the systematic inability of computational systems to extract, represent, and reason over narrative structure and meaning as coherent wholes [12,13]. Existing systems exhibit this limitation by failing to read health information as stories, which can obscure clinically harmful behaviors embedded within otherwise plausible or factually accurate narratives. Our previous work demonstrated that TF-IDF baselines achieved only 12% recall for misinformation detection [14], while an integrated knowledge graph-large language model (LLM) approach reached 72% recall [15]. Even state-of-the-art biomedical transformers such as BioBERT (Bidirectional Encoder Representations From Transformers for Biomedical Text Mining) [16], PubMedBERT [17], and ClinicalBERT [18], which excel at entity recognition in formal clinical text, are not designed to read online stories, that is, to track events, actors, and temporal progression as integrated, meaning-bearing narratives.

Recent multitask learning approaches in clinical natural language processing (NLP) show promise for joint entity extraction and relation classification yet remain focused on formal electronic health records rather than informal, experience-driven health discourse online. Graph-based representations of medical events capture local entity relationships but lack explicit causal directionality and interpretive framing, both essential for understanding narrative intent and credibility. Health misinformation detection systems [19] achieve high accuracy on fact-verification benchmarks but operate at the level of isolated claims, rendering them incapable of distinguishing safe from harmful narratives built from otherwise accurate information. Systems excelling at detecting key phrases or discrete entities fail to capture the causal structure of *who did what* and *what resulted*, producing “context-collapsed” output that loses narrator identity, relationship to events, and emotional framing. Traditional bag-of-words features and sentence-level classifiers cannot encode temporally ordered agent-action-outcome sequences.

The danger becomes concrete when examining actual discussions. A substantial portion of genuinely risky content uses medically accurate terminology while describing clinically harmful behaviors, for example, “Keeping blood sugar stable matters. I’m on a regular 20 units insulin schedule, but I’ve started spacing out doses on days I feel fine because the cost adds up.” Every keyword checks out “blood sugar,” “insulin,” “20 units,” yet this narrative describes potentially fatal self-medication that could precipitate diabetic ketoacidosis. Binary fact-checking systems verify the medical terminology and classify the content as accurate. Narrative-aware systems see a patient rationalizing harmful decision-making wrapped in relatable health care cost concerns. This represents a particularly risky category of health misinformation, content that is largely invisible to keyword-based and semantic similarity approaches because individual facts are correct even as narrative logic is lethal.

This paper introduces the Narrative Builder, which reconstructs unstructured health text into structured agent-action-outcome (AAO) narrative graphs enriched with emotion, stance, and tone metadata. By reconstructing causal experiences embedded in stories, the approach produces machine-readable representations that preserve contextual meaning humans use to interpret health information. Unlike neural event-chain models [20,21] that learn sequences through unsupervised mining, we operationalize established sociolinguistic and psychological theory. Where event-chain models represent events as verb argument structures, the AAO schema distinguishes agents, actions, and outcomes with explicit causal directionality and interpretive dimensions, producing structured graphs suitable for downstream medical verification.

### Study Objectives

This study addresses these challenges through integrated theoretical and empirical contributions with 3 specific aims. First, we formalize narrative blindness as a measurable property of NLP systems, defining 5 measurable dimensions that build on the research gaps surveyed by Ranade et al [12]. Second, we develop a dual-model extraction pipeline integrating Labov and Waletzky’s sociolinguistic narrative theory [13] with biomedical transformers and established interpretive frameworks from psychology [22,23] and clinical communication [24,25], creating a system that reconstructs unstructured health text into AAO narrative graphs enriched with interpretive dimensions (emotion, stance, and tone). Third, through rigorous empirical validation using 5386 Reddit health threads with expert annotation, we demonstrate that theory-grounded architectures produce coherent, medically plausible outputs suitable for downstream credibility assessment. We introduce AAO narrative graphs as a novel representation format bridging computational models with human narrative understanding. We examine whether multitask learning across narrative dimensions provides performance benefits over single-task approaches and whether explicit causal direction detection improves representation of health decision-making compared with linear event models.

## Methods

### Study Design

#### Overview

This study uses a mixed methods research design integrating qualitative narrative theory with quantitative natural language processing [26]. Building on the research gaps surveyed by Ranade et al [12], we define and operationalize narrative blindness as 5 measurable dimensions formalized for health discourse in this work: structural blindness (missing story organization), agentive blindness (entities without narrative roles), causal blindness (correlation without causation), temporal blindness (sequence without temporal meaning), and interpretive blindness (facts without framing). The Narrative Builder operationalizes all 5 dimensions within the health domain.

Figure 1 provides the theoretical foundation, mapping Labov and Waletzky’s narrative components to the computational AAO schema and indicating where interpretive dimensions (emotion, stance, and tone) are most prominently expressed. The overall system architecture is illustrated in Figure 2, showing the dual-transformer pipeline that processes narrative text through interpretive classification (DistilBERT) and structural extraction (BioBERT) to produce AAO narrative graphs.

**Figure 1.**
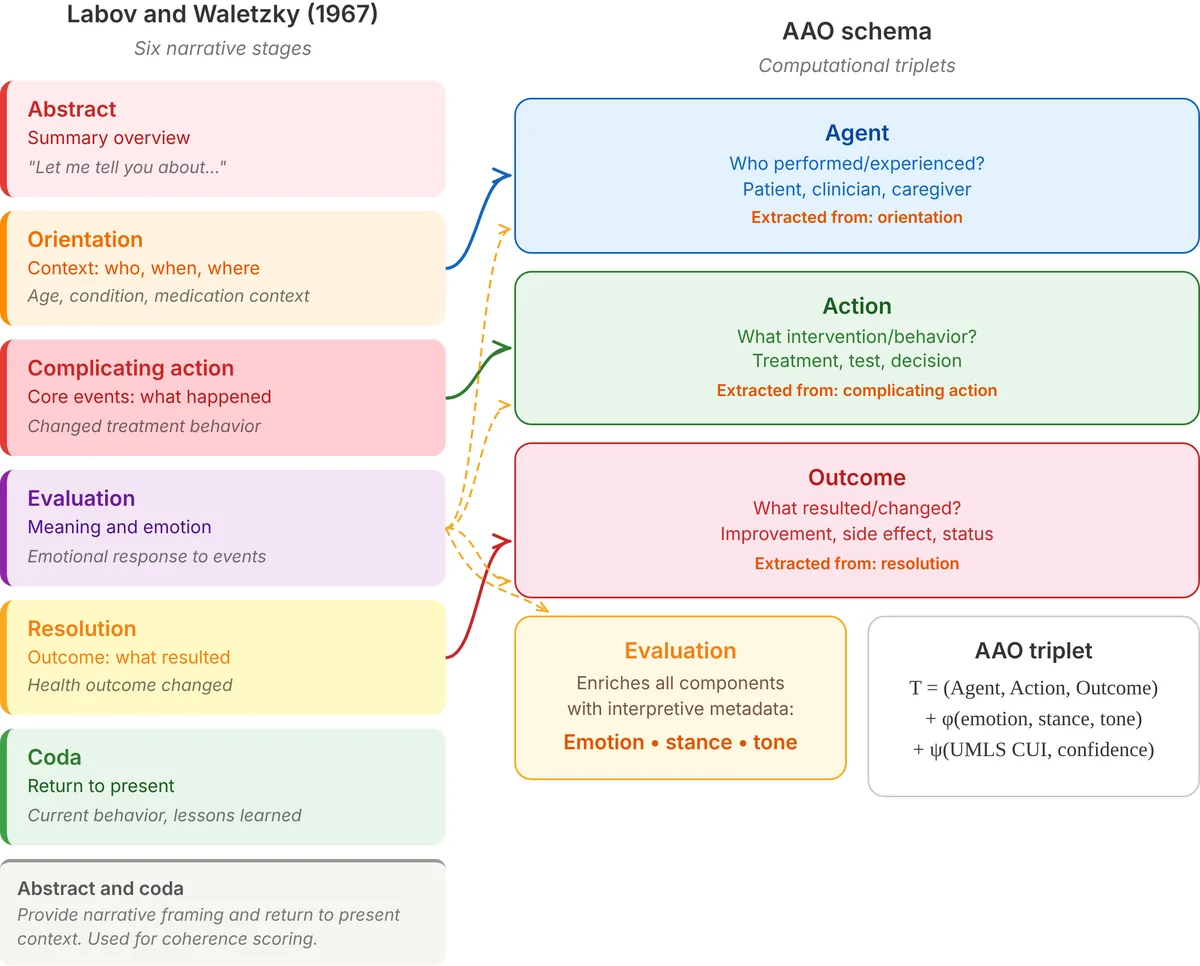
Mapping Labovian narrative structure to agent-action-outcome (AAO) schema. Conceptual diagram showing how Labov and Waletzky’s 6 narrative components [13,27] (abstract, orientation, complicating action, evaluation, resolution, and coda) correspond to AAO triplets. Solid arrows indicate which stages contain agents, actions, and outcomes. Dashed arrows show where interpretive dimensions (emotion, stance, and tone) from the evaluation stage enrich all AAO components. CUI: concept unique identifier; UMLS: Unified Medical Language System.

**Figure 2.**
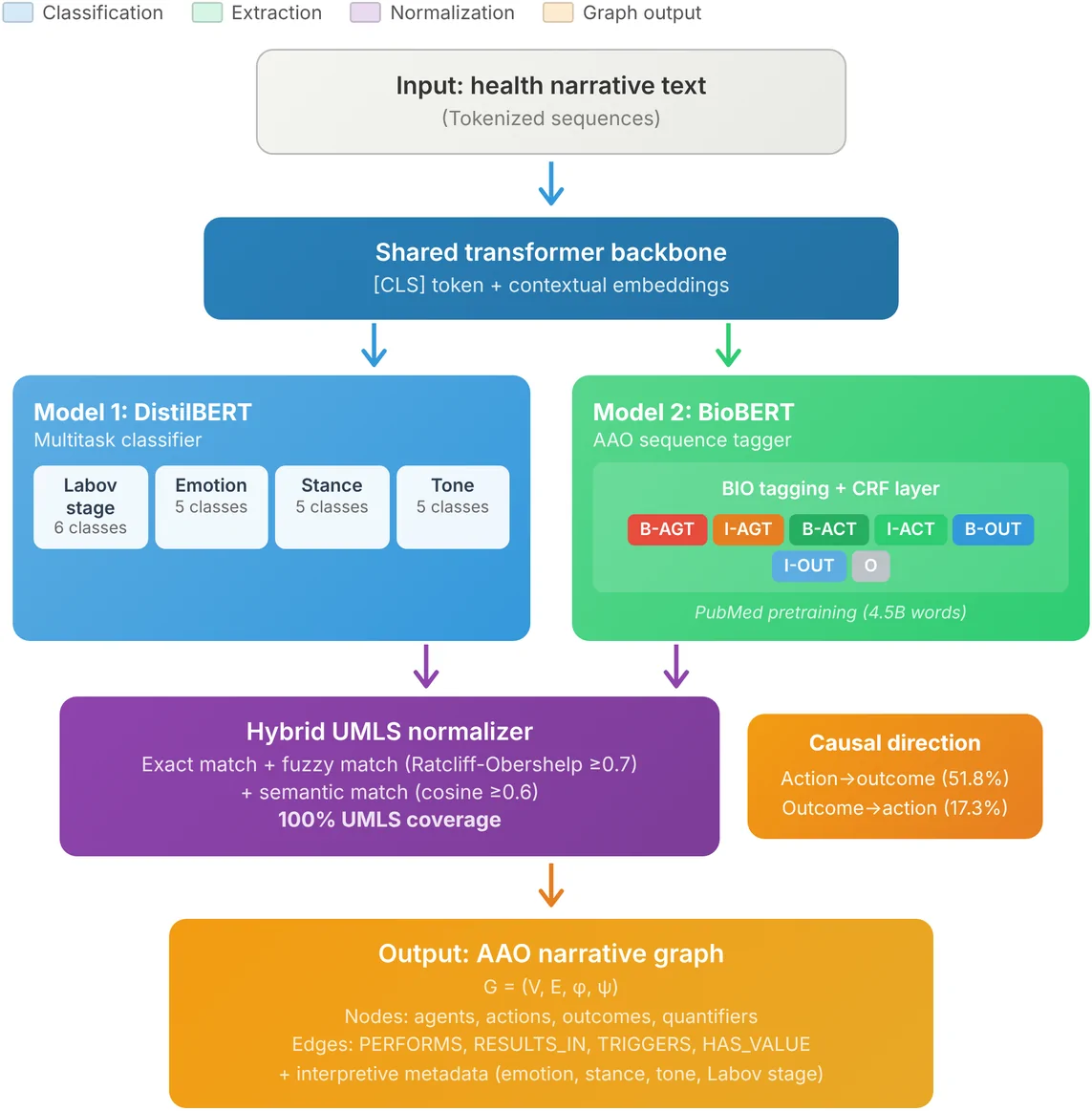
Dual-transformer Narrative Builder architecture. Two-stage pipeline: (1) Multitask DistilBERT classifying Labovian stage, emotion, stance, and tone; (2) BioBERT (Bidirectional Encoder Representations From Transformers for Biomedical Text Mining) performing BIO (beginning-inside-outside) tagging for agent-action-outcome (AAO) extraction with UMLS normalization. Outputs combine into AAO triplets with causal direction detection (action-to-outcome vs outcome-to-action) and clinical quantifier extraction. Addresses five narrative blindness dimensions: structural, agentive, causal, temporal, and interpretive. CRF: conditional random field; UMLS: Unified Medical Language System.

#### Formalizing Narrative Blindness

In this study, narrative blindness is formally defined as the systematic inability of computational systems to extract, represent, and reason over the structural, causal, and interpretive dimensions of narratives, resulting in context-collapsed outputs that lose narrator identity, relationships to events, and emotional framing.

We operationalize narrative blindness through 5 measurable gaps, each corresponding to specific evaluation metrics. Structural blindness reflects the inability to segment narratives into functional components such as abstract, orientation, complicating action, evaluation, resolution, and coda. Agentive blindness occurs when systems extract entities without distinguishing narrative roles, failing to identify who performs actions vs who experiences outcomes. Causal blindness manifests as recognition of co-occurrence without directionality, preventing determination of whether actions cause outcomes or outcomes trigger actions. Temporal blindness involves ordering events without representing temporal meaning, conflating sequence with causation. Interpretive blindness processes factual content without capturing emotional framing, stance, or persuasive intent.

### Theoretical Framework

#### Overview

The framework is grounded in Labov and Waletzky’s sociolinguistic theory of oral narratives [13,27], which identifies 6 structural components: abstract, orientation, complicating action, evaluation, resolution, and coda. How these components align with AAO extraction and where interpretive dimensions are most prominently expressed are shown in Figure 1.

This framework is well suited to health narratives, where evaluation stages often reveal how clinical events are framed as emotionally meaningful experiences that influence decision-making, while orientation establishes participant roles and situational context affecting causal interpretation. For example, narratives describing “reducing insulin” differ substantially depending on whether the agent is newly diagnosed or managing long-term complications.

Labov’s framework was selected over alternatives such as Rhetorical Structure Theory (RST) [28] because health narratives are predominantly experiential rather than argumentative. Labov explicitly models evaluation, capturing emotional framing central to credibility assessment, whereas RST focuses on document-level coherence relations more appropriate for argumentative text.

Emotion categories (fear, hope, frustration, relief, anger) were adapted from Plutchik’s psychoevolutionary theory [22,23]. Stance categories (compliant, resistant, questioning, advocating, neutral) were drawn from medical adherence literature [24,29], and tone categories (clinical, anxious, confident, supportive, skeptical) from clinical communication research [25,30]. Full operational definitions are provided in Multimedia Appendix 1.

#### Agent-Action-Outcome Schema

Narrative understanding is operationalized through AAO triplets, extending event extraction paradigms [20,21] with explicit causal structure. Each narrative event is encoded as *T* = (agent, action, outcome), where the agent initiates or experiences an action, the action represents an intervention or behavior, and the outcome denotes the observable result. Unlike Abstract Meaning Representation (AMR) [31] or PropBank [32], the AAO schema explicitly encodes causal directionality and medical outcome attribution, distinguishing action-to-outcome patterns from outcome-to-action patterns critical for health risk assessment. Figure 1 illustrates the theoretical alignment between Labovian stages and AAO components.

### Data Collection

Reddit was selected based on established criteria for social media health research [5,33]. The platform’s pseudonymous structure encourages candid health disclosure, and its long-form conversational style supports narrative-rich content, with a mean post length of 238.6 (SD 182.4) tokens (Table 1). Community-based moderation provides baseline quality control, and publicly accessible data via the official API enables compliant data collection.

**Table 1.** Corpus characteristics and annotated subset.^a^

Characteristic	Full corpus	Annotated subset
Threads	5386 threads	704 threads
Comments	185,181 comments	2000 segments
Text volume	2,295,242 words	82,826 annotated tokens
Average comments/thread, mean (SD)	34.4 (47.2)	34.1 (45.8)
Average post length (tokens), mean (SD)	238.6 (182.4)	241.2 (179.1)
Date range	February 2019-November 2024	Stratified sample
Interrater reliability (α)	—^b^	0.75‐0.82

a Descriptive statistics for 5386 Reddit health threads (February 2019-November 2024) and 2000 expert-annotated narrative segments. Interrater reliability (Krippendorff α) ranged from 0.75 to 0.82 across all annotated tasks (0.78 to 0.81 for the 4 interpretive classification tasks). In the annotated subset column, threads, average comments/thread, and average post length describe the 704 source threads; the comments row reports the 2000 annotated segments, and the token total covers annotated segments only.

b Not applicable.

Using PRAW v7.6.0, we collected 5386 threads spanning February 2019 through November 2024, comprising 185,181 comments and 2,295,242 words. The collection used 2 strategies. First, 2000 high-engagement threads were retrieved by keyword search (diabetes, obesity, hypertension, ivermectin, and high blood pressure) across r/AskDocs, r/Coronavirus, r/COVID19, r/health, r/medicine, r/nutrition, and r/science; within this batch, the collection keywords were distributed as diabetes 29.0%, obesity 24.9%, hypertension 21.9%, ivermectin 16.6%, and high blood pressure 7.7%. Second, 1185 threads were collected directly from r/diabetes, r/fitness, r/health, r/hypertension, and r/nutrition. A further 2201 threads with extended narrative content were drawn from the same health-focused sources; subreddit-level provenance was not retained for this batch. These keyword proportions describe the first collection batch only and are not a topic-model classification of the full corpus. Table 1 summarizes corpus characteristics.

### Annotation Protocol

#### Annotators and Training

Two domain experts conducted annotations: a biomedical researcher (PhD, neuroimmunology, 25 years pharmaceutical research, and drug development) and a medical doctor (18 y clinical practice). Training included a calibration phase (n=100 segments) with iterative guideline refinement and adjudication sessions to harmonize boundary decisions.

#### Procedure

A 2-stage protocol was applied. In stage 1, annotators assigned Labovian stage labels at the sentence level. In stage 2, annotators labeled AAO spans using beginning-inside-outside (BIO) tagging, linking spans to maintain narrative continuity. Ambiguous agency defaulted to the most plausible actor, and mixed emotional content was resolved by selecting the dominant emotion. Full guidelines are provided in Multimedia Appendix 1.

#### Reliability

Interrater reliability (Krippendorff α) was 0.81 for Labovian stages, 0.79 for emotion, 0.78 for stance, and 0.78 for tone, exceeding accepted thresholds [34]. Disagreements in 246 out of 2000 (12.3%) segments were resolved through adjudication. The 2000 annotated segments were drawn from 704 annotated narratives, each corresponding to one thread in the annotation lineage (hereafter, threads). Each thread’s narrative was segmented, and up to 3 most informative segments per thread were retained for annotation: 643 narratives contributed 3 segments, 10 contributed 2, and 51 contributed 1, totaling 2000. The grouping field used for cross-validation, comment_id, partitions the 2000 annotated segments into 704 disjoint groups (mean 2.84, SD 0.53 segments per group); no group is shared across the held-out split or any of the 5 cross-validation folds. Of these segments, 1992 out of 2000 (99.6%) yielded at least one AAO span, and 1625 out of 2000 (81.2%) contained complete AAO structures. Narrative graphs are generated at the thread level, with each of the 704 threads aggregating the AAO triplets from its constituent segments. Figure 3 presents the data flow from collection through annotation to evaluation.

**Figure 3.**
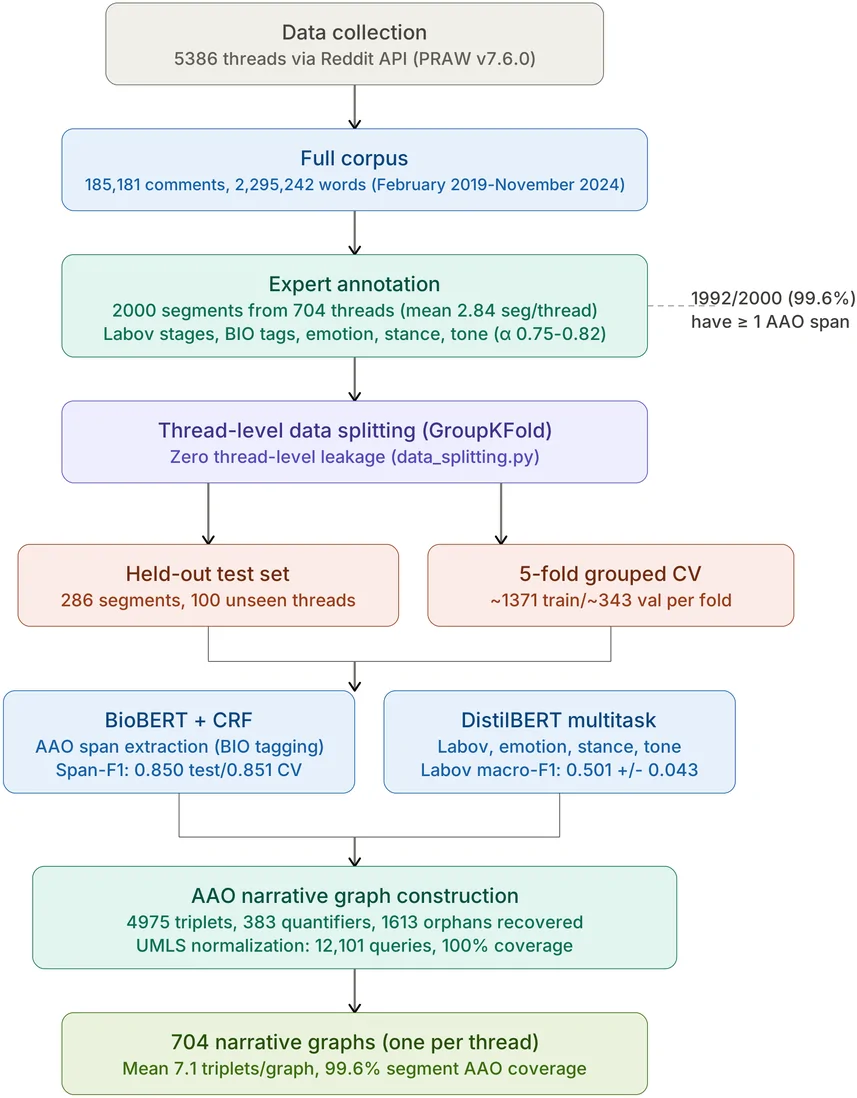
Data flow diagram. Pipeline from 5386 collected threads through expert annotation (2000 segments from 704 threads), grouped splitting on the annotation-level key (GroupKFold on comment_id, zero leakage), dual-model processing (BioBERT+CRF span-*F*_1_=0.850; DistilBERT macro-*F*_1_=0.501), and graph construction (4975 AAO triplets, 704 narrative graphs, 99.6% segment AAO coverage). AAO: Agent-Action-Outcome; BIO: beginning-inside-outside (tagging scheme); BioBERT: Bidirectional Encoder Representations From Transformers for Biomedical Text Mining; CRF: conditional random field; CV: cross-validation; UMLS: Unified Medical Language System.

### Dual-Model Architecture

The Narrative Builder uses 2 transformers in sequence: DistilBERT for multitask narrative classification and BioBERT for medically grounded entity extraction. Figure 2 presents the complete architecture.

#### Multitask Narrative Classification

DistilBERT predicts Labovian stages, emotion, stance, and tone from shared representations [35]. Loss weights were assigned manually to modestly upweight the primary Labovian-stage task (Labov 1.0; emotion, stance, and tone 0.8 each; Multimedia Appendix 2, Table A2.3); no automated search over loss weights was performed. Multitask learning enables shared representations to capture interdependencies between narrative dimensions, such as emotional language clustering in evaluation segments.

#### Entity Extraction and Medical Grounding

The second stage uses BioBERT to extract structural triplets through BIO sequence tagging, identifying which text spans represent agents, actions, and outcomes [12]. BioBERT’s extensive pretraining on 4.5 billion words of biomedical literature provides robust recognition of medical terminology even in informal, colloquial language patients use online. A conditional random field (CRF) layer atop BioBERT enforces logical constraints on tag sequences, preventing impossible patterns like isolated “inside-entity” tags appearing without corresponding “beginning-entity” markers [36].

#### Medical Concept Normalization

Extracted spans are normalized to standardized medical concepts using the UMLS 2024AA Metathesaurus [37] through a 3-stage cascade: exact string matching, fuzzy string matching (Ratcliff-Obershelp similarity ≥0.7, difflib.SequenceMatcher), and BioBERT embedding cosine similarity (threshold ≥0.6). This architecture achieved 100% concept coverage across 12,101 normalization queries in the canonical pipeline run.

#### Causal Direction Detection

Explicit causal directionality is detected to distinguish action-to-outcome from outcome-to-action narratives. Signals include linguistic markers, temporal ordering, emergency indicators, and the co-occurrence of an emergency indicator with a critical clinical value [38]. Implementation details are provided in Multimedia Appendix 3. Directional modeling ensures that narrative graphs represent causal logic accurately, as illustrated in Figure 4.

**Figure 4.**
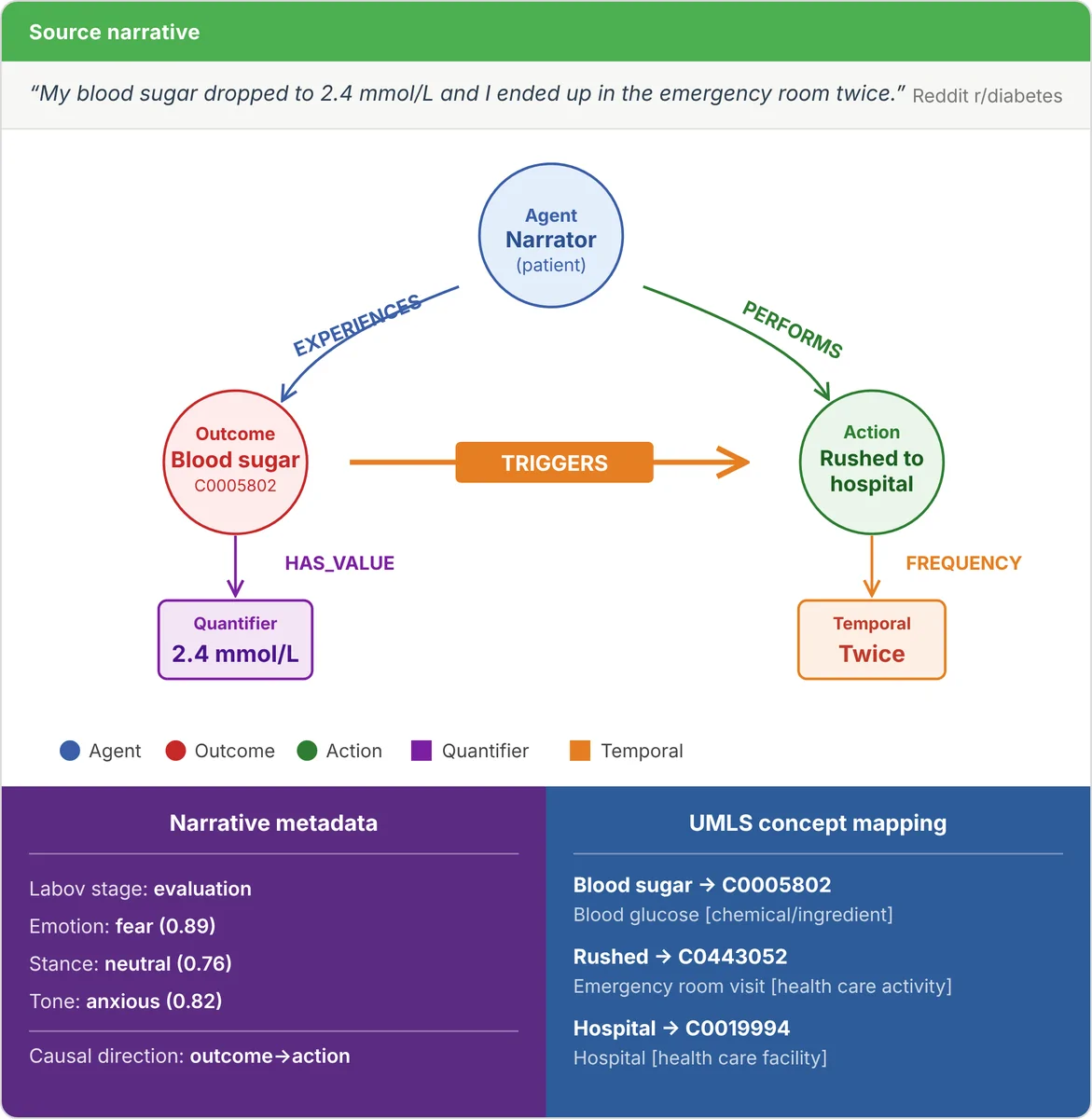
Example agent-action-outcome (AAO) narrative graph for emergency hypoglycemia. Graph representation of a paraphrased Reddit health narrative describing dangerously low blood sugar prompting emergency medical care. Nodes show Unified Medical Language System (UMLS)–mapped entities (agent, outcome, action, quantifier, and temporal); edges show relationships (EXPERIENCES, PERFORMS, TRIGGERS, HAS_VALUE, and FREQUENCY). Panels report the narrative metadata with classifier confidence scores and the UMLS concept mappings. The TRIGGERS edge marks an emergency response (outcome-to-action), as distinct from treatment progression (action-to-outcome).

#### Clinical Quantifier Extraction

Clinical quantifiers (eg, blood glucose, blood pressure, A_1C_, and medication dosages) are extracted and linked to outcomes via HAS_VALUE edges, enabling verification against clinical thresholds. Figure 4 demonstrates this linkage in a complete narrative graph.

#### Implicit Relationship Recovery

Implicit causal relationships are recovered using proximity-based heuristics, reflecting pragmatic causal inference in informal discourse [39]. Outcomes not linked to an agent-action pair are assigned to the narrator by default and linked to the nearest action by token distance, in either direction, within a 100-token window; clinical quantifiers are linked to the nearest outcome within 25 tokens.

### AAO Narrative Graph Construction

AAO triplets are assembled into narrative graphs with nodes, representing UMLS-mapped entities and edges encoding PERFORMS, RESULTS_IN, TRIGGERS, and HAS_VALUE relationships. Each node retains interpretive metadata. Figure 4 presents a complete example.

### Evaluation Framework

Five-fold grouped cross-validation (GroupKFold on comment_id) was used to prevent data leakage across grouping values, ensuring no segments sharing a grouping value appear in both training and evaluation partitions. Metrics included precision, recall, and macro-*F*_1_ for span extraction and per-task macro-*F*_1_ for the interpretive classification tasks. Cross-validation figures are reported as the mean and SD across the 5 grouped folds. Statistical comparisons used McNemar test with continuity correction, applied at the entity-span level on a shared grouped held-out test set (286 segments from 100 unseen threads) [40].

Baseline comparisons included TF-IDF with logistic regression and single-task BERT. ClinicalBERT and PubMedBERT were not treated as baselines because they function as interchangeable encoders within the proposed framework rather than standalone narrative reconstruction systems.

Expert validation involved 2 domain experts rating 100 generated graphs on anchored 0 to 1 continuous scales. The biomedical researcher rated narrative coherence only and the physician rated medical plausibility only; each rated all 100 graphs independently, without consultation. Rating materials and rubrics are provided in Multimedia Appendix 4.

### Ethical Considerations

This study used publicly available Reddit data collected via Reddit’s official API in compliance with Reddit’s terms of service and established guidelines for social media research ethics. No personally identifiable information was collected. The study was determined exempt from institutional review board review under 45 CFR 46.104(d)(4), consistent with research involving publicly available data, which covers secondary research use of publicly available information. All narrative examples in this manuscript have been paraphrased to prevent reidentification. This approach follows established ethical guidelines for social media health research [33,41].

## Results

### AAO Extraction Performance

The Narrative Builder achieved span-*F*_1_ of 0.850 on the grouped held-out test set (mean 0.851, SD 0.007 across 5-fold grouped cross-validation; population SD across folds, ddof=0), significantly outperforming the TF-IDF baseline at 0.781 (*P*<.001; McNemar test). The CRF layer provided a modest, nonsignificant improvement over BioBERT without CRF (0.841; *P*=.27), with its primary value being structural enforcement of valid BIO transition constraints through a learned transition matrix (Table 2).

**Table 2.** Comparative performance of agent-action-outcome (AAO) extraction models.^a^

Model	Span-*F*_1_	McNemar *P* (vs CRF^b^)
TF-IDF^c^ + logistic regression	0.781	<.001
BioBERT^d^ (no CRF)	0.841	.27
BioBERT + CRF (proposed)	0.850	Reference

a Three systems compared on the same grouped held-out test set (286 segments, 100 unseen threads, 2138 gold entity spans). *P* values from the McNemar test with continuity correction. Gold span counts differ across systems because tokenization differs; the TF-IDF pipeline is scored against 2267 gold spans, and the 2 transformer systems against 2138. McNemar tests operate on the 2138 aligned spans.

b CRF: conditional random field.

c TF-IDF: term frequency-inverse document frequency.

d BioBERT: Bidirectional Encoder Representations From Transformers for Biomedical Text Mining.

Component-level performance varied: action spans achieved the highest *F*_1_ (0.90; precision=0.947; recall=0.857), followed by agent (0.83; precision=0.890; recall=0.787) and outcome (0.77; precision=0.790; recall=0.750). Agent detection was challenged by implicit self-reference where patients omit pronouns, while outcome extraction was the most difficult component, reflecting the diverse linguistic forms outcomes take in informal health discourse (Table 3).

**Table 3.** Per-type span-level precision, recall, and *F*_1_-score of BioBERT with a conditional random field layer on the grouped held-out test set (286 segments from 100 unseen threads; 2138 gold entity spans).

Type	Precision	Recall	*F*_1_
Action	0.947	0.857	0.900
Agent	0.890	0.787	0.835
Outcome	0.790	0.750	0.770
Micro	0.892	0.812	0.850

Together, these results correspond to performance across the 5 operational dimensions of narrative blindness—structural, agentive, causal, temporal, and interpretive, providing empirical coverage of the full framework defined in *Methods*.

### Narrative Stage Classification

Labovian stage classification achieved mean macro-*F*_1_ of 0.501 (SD 0.043) across 5-fold grouped cross-validation (Table 4). Performance varied across stages, with complicating action and resolution achieving the strongest classification due to distinctive lexical markers. Orientation leveraged temporal and spatial cues. Abstract and coda exhibited greater variability, often blending with adjacent stages in informal health discourse. Abstracts frequently merged with orientations when narratives began with contextualizing statements rather than explicit summaries. Codas were often absent in Reddit comments, which typically end with questions rather than formal narrative closure. The moderate overall performance reflects the inherent difficulty of classifying informal online discourse into Labovian categories originally developed for oral narrative.

**Table 4.** Multitask DistilBERT classification performance.^a^

Task	Macro-*F*_1_, mean (SD)	Micro-*F*_1_, mean (SD)
Labov stage	0.501 (0.043)	0.609 (0.028)
Stance	0.528 (0.036)	0.655 (0.032)
Emotion	0.466 (0.032)	0.578 (0.016)
Tone	0.465 (0.023)	0.589 (0.027)

a Task-level macro-*F*_1_ for Labovian stage classification and 3 interpretive dimensions, evaluated on 2000 annotated segments using 5-fold grouped cross-validation (GroupKFold on comment_id).

### Interpretive Dimension Classification

Interpretive classification achieved the following performance across 5-fold grouped cross-validation: emotion (mean macro-*F*_1_ 0.466, SD 0.032), stance (mean macro-*F*_1_ 0.528, SD 0.036), tone (mean macro-*F*_1_ 0.465, SD 0.023).

Emotion classification was the most challenging interpretive dimension (mean macro-*F*_1_ 0.466, SD 0.032), reflecting the difficulty of distinguishing emotional states in informal text where multiple emotions co-occur and markers overlap (eg, frustration and anger in health care complaints). Stance classification achieved the highest interpretive performance (mean macro-*F*_1_ 0.528, SD 0.036), with the classifier better able to distinguish compliance and resistance markers that carry distinctive lexical signals. Tone classification (mean macro-*F*_1_ 0.465, SD 0.023) proved similarly challenging, with overlapping markers where uncertainty and conviction coexist in health narratives.

### Multitask Learning Ablation Study

We compared single-task and multitask DistilBERT configurations across the 4 classification tasks (Table 5). The single-task configuration achieved higher macro-*F*_1_ on 3 of 4 tasks compared to multitask training. We therefore do not claim a multitask performance benefit; the shared encoder architecture is retained for computational efficiency (one forward pass for 4 tasks) rather than for performance gains. The interpretive dimensions (emotion, stance, and tone) provide contextual metadata that enriches the AAO graph with narrative framing, independent of whether joint training improves individual task performance.

**Table 5.** Single-task vs multitask DistilBERT comparison.^a^

Task	Multitask *F*_1_	Single-Task *F*_1_	Delta	95% CI (Δ)	Paired *t* test, *t* (*df*); *P* value
Labov	0.493	0.516	−0.023	−0.060 to +0.013	–1.75 (4); .15
Emotion	0.493	0.487	+0.005	−0.066 to +0.077	0.20 (4); .85
Tone	0.490	0.507	−0.017	−0.081 to +0.047	–0.73 (4); .50
Stance	0.549	0.572	−0.024	−0.057 to +0.010	–1.98 (4); .12

a Matched-fold paired comparison using identical encoder, hyperparameters, and grouped folds. Single-task trains one model per task; multitask trains all 4 heads jointly. No significant multitask benefit on any task (all *P*≥.12). 95% CIs are paired *t* intervals on the mean per-fold delta (multitask minus single-task, 5 grouped folds); every interval includes zero. All t tests are 2-tailed paired *t* tests (*df*=4).

### Production Pipeline Statistics

Pipeline execution across 5386 threads generated 4975 AAO triplets from 704 threads (mean 7.1, SD 3.1 triplets per thread). UMLS normalization achieved 100% coverage across 12,101 normalization queries, resolved through a 3-stage cascade: SQLite exact matching (7769/12,101, 64.2%), SQLite fuzzy matching (3185/12,101, 26.3%), and BioBERT semantic matching (1147/12,101, 9.5%; mean cosine similarity 0.9499, SD 0.0182). Causal direction analysis at the segment level (N=2000) identified 1035 (51.8%) action-to-outcome patterns, 346 (17.3%) candidate outcome-to-action patterns, 155 (7.8%) bidirectional relationships, and 464 (23.2%) undetermined. A targeted validation on 143 hand-labeled segments found the rule-based causal classifier achieved overall accuracy of 0.371 (53/143), with outcome-to-action precision of 0.277 (13/47; 95% CI 0.149-0.404). Clinical quantifier extraction identified 383 measurements from 64 narratives, including 2 emergency-level critical values. Validation on a stratified sample yielded precision of 0.900 (108/120; 95% CI 0.842-0.950) and recall of 0.604 (29/48; 95% CI 0.458-0.729). Clinical severity thresholds follow published clinical guidelines: glucose and HbA_1c_ thresholds are based on the American Diabetes Association Standards of Care in Diabetes-2024 [42] (glucose: severe hypoglycemia <2.8 mmol/L, set below the ADA level 2 threshold of 3.0 mmol/L, hyperglycemia >11.1 mmol/L; HbA_1c_: diabetes ≥6.5%), and blood-pressure severity tiers follow the 2017 ACC/AHA hypertension guideline [43] (hypertensive crisis ≥180/120 mm Hg, stage 2 ≥140/90, stage 1 ≥130/80, elevated ≥120 systolic). These enable verification against clinical thresholds, distinguishing qualitative descriptions from verifiable assertions (Figure 4).

### Expert Validation of Narrative Graphs

Two domain experts independently evaluated 100 randomly selected narrative graphs using anchored 0 to 1 continuous scales. The biomedical researcher assessed narrative coherence, rating whether graphs preserved logical flow and causal relationships of original narratives. The practicing physician assessed medical plausibility, rating whether extracted entities and relationships aligned with clinical knowledge.

Narrative coherence achieved mean rating of 0.89 (SD 0.08), indicating that the majority of generated graphs successfully preserved narrative structure. Medical plausibility achieved mean rating of 0.91 (SD 0.07), indicating strong alignment with clinical knowledge (see *Limitations* regarding potential confirmation bias). The 2 dimensions were assessed independently by raters with distinct expertise; because the raters scored different constructs (coherence vs plausibility), no cross-rater agreement statistic is defined or reported.

Qualitative analysis of the 9 lowest-scoring graphs revealed 2 primary failure modes. The first involved incorrect causal direction assignment in narratives containing ambiguous temporal markers where the relationship between events could not be definitively established from linguistic context alone. For example, “My blood sugar reading was very low so I grabbed some glucose right away” could represent either emergency response (outcome-to-action) or preventive action (action-to-outcome) depending on implicit context. The second failure mode consisted of missed implicit agent references in passive constructions where the subject performing the action was grammatically omitted, such as “Was prescribed metformin” without clear indication whether the narrator or another patient was the recipient. These failure modes represent targets for future refinement of the causal direction detector and orphan recovery algorithms.

## Discussion

### Principal Findings

The results of this study help explain why online health misinformation remains difficult to detect despite advances in language modeling and biomedical knowledge integration. Our findings indicate that the primary limitation is not one of insufficient accuracy or domain knowledge but of representational scope. Systems optimized for identifying isolated claims or entities struggle to interpret health information expressed as coherent narratives, where meaning emerges from relationships among actors, actions, outcomes, and emotional framing rather than from individual facts alone. From an information systems perspective, this reflects a mismatch between how health information is produced and how it is computationally assessed: users communicate health experiences through stories that encode causality, interpretation, and affect, whereas most automated systems decompose these stories into decontextualized features. Our results indicate that reducing narratives to claims or entities alone is insufficient for credibility assessment in health contexts.

Within this representational gap, our findings clarify why clinically harmful content may evade existing detection approaches. We observed that narratives describing unsafe health behaviors can use correct medical terminology and align superficially with clinical language yet convey risky decision-making when interpreted in context. When narrative structure and causal directionality are not explicitly modeled, such content may appear benign to fact-centric systems despite posing genuine health risks. These observations provide empirical grounding for the concept of narrative blindness and motivate the need for narrative-aware computational representations.

We further demonstrate that theory-grounded transformer architectures can overcome narrative blindness through structured reconstruction of health narratives. By formalizing narrative blindness, building on the research gaps surveyed by Ranade et al [12], and integrating Labov’s sociolinguistic theory [13,27] with interpretive frameworks from psychology [22,23] and clinical communication [24,25], we achieved 3 findings aligned with our study objectives. First, narrative blindness was formalized as a measurable construct through 5 operational gaps, enabling systematic evaluation of how existing systems fail to capture narrative dimensions. Second, the dual-model Narrative Builder achieved span-*F*_1_=0.850 for AAO extraction, outperforming the TF-IDF baseline (span-*F*_1_=0.781; *P*<.001; McNemar test). Labovian stage classification reached macro-*F*_1_=0.501, reflecting the inherent difficulty of categorizing informal online discourse into sociolinguistic stages. Third, independent expert review confirmed that generated narrative graphs preserve narrative coherence (mean 0.89, SD 0.08) and medical plausibility (mean 0.91, SD 0.07), supporting suitability for downstream credibility assessment.

The multitask architecture provides computational efficiency through shared representations, though single-task configurations achieved comparable or higher performance on individual classification tasks. Performance across narrative dimensions demonstrated the feasibility of structured narrative reconstruction, including structural (AAO span-*F*_1_=0.850), agentive (AGENT span-*F*_1_=0.835), and causal direction detection (51.8% action-to-outcome, 17.3% candidate outcome-to-action, with measured limitations in causal classification accuracy). Interpretive dimensions achieved moderate performance (emotion 0.466; stance 0.528; tone 0.465), establishing baselines for future refinement. The pipeline identified 17.3% of segments as candidate inverted causality patterns (outcome triggering action), though validation indicated limited precision for this heuristic (precision=0.277). Nevertheless, the structural capability to represent bidirectional causality in AAO graphs, as demonstrated qualitatively in Figure 4, addresses a representational gap absent from existing event-chain models.

### Comparison With Existing Approaches

These findings provide an empirical instantiation of the narrative-understanding gaps surveyed by Ranade et al [12] through computational implementation, demonstrating that bag-of-words and sequence models fail to capture narrative meaning when evaluated against structured narrative criteria. The significant improvement over the TF-IDF baseline (span-*F*_1_ 0.850 vs 0.781; *P*<.001) indicates that narrative reconstruction benefits from contextual language models capable of capturing relationships across longer spans, though the CRF contribution beyond BioBERT alone was modest (*P*=.27).

The AAO schema advances beyond existing semantic representation frameworks by explicitly encoding causal directionality essential for health risk assessment. AMR [31] and PropBank [32] capture general semantic and verb-argument structure but do not distinguish action-to-outcome from outcome-to-action patterns. This limitation is relevant to our corpus, where the pipeline identified 17.3% of segments as candidate emergency-response patterns. For example, interpreting “My blood sugar was 2.4 mmol/L so I rushed to the hospital” requires recognizing that a harmful outcome triggered action, rather than the reverse. AMR and PropBank lack mechanisms for encoding this clinically meaningful causal attribution.

Biomedical NLP systems such as BioBERT [16], PubMedBERT [17], and ClinicalBERT [18] achieve strong performance on entity extraction from formal clinical text but are not designed to reconstruct narrative structure in informal online discourse. These models excel at recognizing medical terminology yet lack representations for narrative progression, agency, and contextual meaning embedded in lived experience. Our framework complements biomedical encoders by providing narrative scaffolding that situates extracted entities within causally structured patient stories. BioBERT serves as the entity extraction backbone precisely because of its clinical strength, while the Narrative Builder extends it with Labovian stage modeling, multitask interpretive dimensions, and explicit causal reasoning.

Event-chain models [20,21] learn probabilistic event sequences through unsupervised mining, representing events as verb-argument structures without narrative role differentiation. While effective for identifying common event patterns, these approaches assume linear temporal progression, lack bidirectional causality modeling, omit interpretive dimensions, and operate without clinical grounding. In contrast, our theory-grounded framework integrates structural, causal, and interpretive dimensions within graph-based representations normalized to medical ontologies, enabling systematic verification and narrative-level reasoning.

Health misinformation detection research has largely focused on binary classification of isolated claims [19,44]. Although such systems perform well on fact-verification benchmarks, they lack mechanisms for contextual risk assessment. Approaches trained on COVID-19 misinformation datasets [44,45] detect false claims but cannot distinguish safe from harmful uses of accurate information. A statement such as “insulin affects blood sugar” is classified as accurate regardless of whether it appears in clinical guidance or in a narrative advocating unsafe self-medication. Knowledge graph-LLM hybrids [15] improve entity-level verification but still treat health information as collections of facts rather than causally structured narratives. Our framework differs by preserving narrative context, AAO relationships, and emotional framing that determine whether accurate information is presented safely or harmfully.

While the multitask architecture did not yield statistically significant performance improvements over single-task configurations, the shared encoder provides computational efficiency and produces the interpretive metadata (emotion, stance, tone) that enriches AAO graphs with narrative context.

### Limitations

This study has several limitations that should inform interpretation of the findings.

First, the dataset is restricted to English-language Reddit discussions. Reddit’s long-form conversational style differs from short-form platforms such as Twitter, multimedia-heavy platforms like TikTok, and social network sites like Facebook. Cross-platform generalization will require validation and platform-specific adaptation. Second, the current implementation is optimized for single-narrator accounts with relatively unambiguous agency. Multiparty dialogues and threaded conversations present challenges requiring discourse modeling and coreference resolution beyond the present scope. Third, although interrater reliability was acceptable (α=0.75‐0.82 across all annotated tasks), annotation involves subjective judgments regarding narrative stages and emotional intensity, with 246 out of 2000 (12.3%) segments requiring adjudication. Fourth, the rule-based causal direction classifier achieved limited accuracy (0.371 overall, with outcome-to-action precision of 0.277). The causal direction distribution reported in *Results* should be interpreted as candidate detections from an exploratory heuristic rather than validated classifications. Fifth, the interpretive dimension classifiers achieved moderate performance (macro-*F*_1_ ranging 0.47‐0.53), reflecting the inherent subjectivity of these dimensions in informal online discourse. These results establish baselines rather than production-ready classification. Sixth, while 99.6% of the 2000 annotated segments yielded at least one AAO span, brief questions, single-word replies, and purely factual exchanges may produce sparse graphs with limited analytical value. Seventh, upstream Reddit submission identifiers were not retained in the annotation file; the grouping key, comment_id, is a reindexed integer key (values 0-703) assigned when the annotation file was constructed. Each grouping value corresponds to one annotated narrative in the annotation lineage, with up to the 3 most informative segments retained per narrative under the segmentation protocol, but the correspondence between grouping values and original Reddit submissions cannot be reverified against platform identifiers; the zero-leakage guarantees therefore hold at the level of this grouping key. Finally, the expert validation of narrative graphs was conducted by the same domain experts who created the gold-standard annotations. While their domain expertise was necessary for evaluating medical plausibility, this overlap may introduce confirmation bias. Each dimension was additionally assessed by a single rater rather than by multiple raters, so no reliability estimate is available for either coherence or plausibility.

Future work should address these limitations through four priorities: (1) independent expert validation with reviewers who did not participate in annotation, (2) a learned causal direction model leveraging the AAO graph structure, (3) larger annotated datasets and few-shot approaches to improve interpretive classification, and (4) cross-platform validation on short-form and multimedia health content.

### Conclusions

This work demonstrates that limitations in health misinformation detection are not solely attributable to insufficient data or model capacity but to how health information is represented and reasoned over computationally. When systems lack the ability to interpret narrative structure and causal relationships, clinically important context may be lost, constraining the effectiveness of existing mitigation approaches.

This study formalizes narrative blindness as a measurable limitation of current computational systems and demonstrates that theory-grounded transformer architectures can address it through structured narrative reconstruction. By integrating sociolinguistic narrative theory with interpretive frameworks from psychology and clinical communication, the Narrative Builder preserves contextual relationships, causal logic, and emotional significance that are essential for evaluating the credibility of health information shared as lived experience rather than isolated factual claims. The resulting AAO graphs provide machine-readable representations suitable for downstream verification systems, enabling context-aware credibility assessment beyond traditional fact-checking approaches.

The significant improvement in AAO extraction over the TF-IDF baseline (span-*F*_1_ 0.850 vs 0.781), together with expert validation of narrative coherence and medical plausibility, indicates that computational systems can reconstruct structured narrative representations from informal health discourse. Explicit modeling of causal directionality, while requiring further refinement of the rule-based classifier, addresses a representational gap in existing approaches, particularly in narratives where outcomes precipitate subsequent actions.

Importantly, these findings have implications beyond technical performance. Online health misinformation frequently manifests as personal stories that embed clinically relevant decisions within emotionally persuasive and causally structured narratives. Systems that are unable to interpret such narratives as coherent wholes risk overlooking harmful content that appears medically plausible at the claim level while conveying unsafe behaviors in context. As a result, narrative blindness may contribute to the persistence of health misinformation that evades existing detection mechanisms, limiting the effectiveness of current public health monitoring and intervention strategies.

By supporting narrative-centered rather than fact-centric analysis, the proposed framework enables more nuanced interpretation of public health discourse and provides a foundation for assessing credibility in ways that better reflect how individuals consume and act upon health information online. While further validation across health domains, populations, and platforms is required, these results suggest that operationalizing narrative structure is a feasible and valuable complement to existing public health text analysis methods, with potential to enhance risk-aware information assessment in real-world settings.

## Supplementary material

10.2196/83496 Multimedia Appendix 1 Annotation guidelines and theoretical grounding.

10.2196/83496 Multimedia Appendix 2 Model hyperparameters, per-type extraction performance, single-task vs multitask comparison, McNemar test results, heuristic component validation (orphan recovery, clinical-value regex, causal direction), and UMLS normalization cascade statistics.

10.2196/83496 Multimedia Appendix 3 Production implementations, reproduced verbatim from the deposited pipeline with source line ranges, of causal direction detection, clinical quantifier extraction and classification, and entity linking with orphan recovery.

10.2196/83496 Multimedia Appendix 4 Expert validation rubric with rating scale anchors and independent single-rater coherence and plausibility assessments.

## Abbreviations

AAO: agent-action-outcome
AMR: Abstract Meaning Representation
BIO: beginning-inside-outside (tagging scheme)
BioBERT: Bidirectional Encoder Representations From Transformers for Biomedical Text Mining
CRF: conditional random field
CUI: concept unique identifier
LLM: large language model
NLP: natural language processing
RST: Rhetorical Structure Theory
TF-IDF: term frequency–inverse document frequency
UMLS: Unified Medical Language System
