# Supplementary material for: AI-Driven Reconstruction of Online Health Narratives Using Agent-Action-Outcome Modeling: Algorithm Development and Validation
Source: JMIR AI. 2026 Nov 13;5:e83496. doi: 10.2196/83496 (PMC13766579; doi:10.2196/83496)
Supplement: Supplementary file 1 [file ai-v5-e83496-s001.pdf]

Multimedia Appendix 1: Annotation Guidelines and Theoretical Grounding

1. Interpretive Dimension Taxonomy

Table: Interpretive Dimension Classification Labels with Theoretical Grounding

Dimension	Framework	Label	Operational Definition	Example from Health Narratives
**Emotion**	Plutchik [22,23]	**Fear**	Expressed anxiety about diagnosis, treatment outcomes, or disease progression	"I'm terrified about what the doctor might find"
		**Hope**	Expressed optimism about recovery, treatment efficacy, or positive outcomes	"I really believe this new medication will help me finally feel better"
		**Frustration**	Expressed irritation with treatment failures, access barriers, or lack of progress	"I've tried everything and nothing works - this is so exhausting"
		**Relief**	Expressed comfort, reassurance, or satisfaction following symptom improvement or good news	"The test came back negative and I can finally breathe again"
		**Anger**	Expressed resentment toward diagnosis, healthcare providers, insurance, or treatment	"My doctor dismissed my concerns completely, I'm furious"
**Stance**	Horne [24], DiMatteo [29]	**Compliant**	Endorses following medical recommendations, expresses intention to adhere	"I take my medication exactly as prescribed every day"
		**Resistant**	Endorses opposing, rejecting, or modifying medical recommendations against advice	"I stopped taking the pills because they made me feel worse"
		**Questioning**	Expresses uncertainty, seeks clarification, or requests second opinion	"Should I really be on this high a dose? I'm not sure"
		**Advocating**	Promotes specific treatments, approaches, or behaviors to other community members	"You should definitely try the keto diet; it changed my life"
		**Neutral**	Reports experience without evaluative positioning toward medical recommendations	"I started metformin last month and my A1C is now 6.5"
**Tone**	Street [25], Epstein & Street [30]	**Clinical**	Uses medical terminology, objective presentation, emotionally neutral register	"Fasting glucose was 8.2 mmol/L; commenced 500mg metformin BID"

		**Anxious**	Expresses worry, seeks reassurance, conveys nervousness or uncertainty	"I'm really scared about this procedure, what if something goes wrong?"
		**Confident**	Asserts advice or describes experience with certainty and self-assurance	"Trust me, I've dealt with this for years, here's what you need to do"
		**Supportive**	Offers encouragement, empathy, or solidarity to other community members	"You've got this! We're all here for you and understand what you're going through"
		**Skeptical**	Expresses doubt about conventional treatments, questions medical authority	"Big Pharma just wants your money, natural remedies are better"

Interpretive taxonomy for emotion, stance, and tone in Reddit health narratives. Categories, theoretical frameworks, and operational definitions used to annotate emotion, stance, and tone in 2,000 narrative segments. Emotion labels are adapted from Plutchik's psychoevolutionary theory; stance labels from healthcare adherence literature; and tone labels from clinical communication research.

2. Labovian Narrative Stage Definitions

Based on Labov & Waletzky [13] and Labov [27]:

Abstract

- **Definition:** Summary overview that encapsulates what the narrative is about
- **Function:** Answers "What is this story about?"
- **Typical position:** Opening sentence(s) of narrative
- **Linguistic markers:** "So here's what happened...", "Let me tell you about...", "TL;DR"
- **Example:** "I had a scary hypoglycemic episode last night"

Orientation

- **Definition:** Contextual grounding establishing who, when, where, and what circumstances
- **Function:** Sets the stage for the complicating action
- **Typical position:** Early narrative, after abstract
- **Linguistic markers:** Temporal ("yesterday", "last month"), spatial ("at the hospital"), participant identification ("my doctor", "I")
- **Example:** "I'm 26, diabetic for 3 years, usually well-controlled on metformin"

Complicating Action

- **Definition:** Core event sequence describing what happened - the narrative crisis
- **Function:** Answers "Then what happened?"
- **Typical position:** Middle narrative, main body
- **Linguistic markers:** Past tense verbs of action ("started", "developed", "experienced"), temporal sequencers ("then", "after that")
- **Example:** "My blood sugar dropped to 2.4 mmol/L and I started shaking uncontrollably"

Evaluation

- **Definition:** Narrator’s interpretation, emotional response, and significance assessment
- **Function:** Answers “So what? Why does this matter?”
- **Typical position:** Interspersed throughout or clustered near climax
- **Linguistic markers:** Evaluative adjectives (“terrifying”, “concerning”), intensifiers (“really”, “so”), emotional expressions
- **Example:** “It was the scariest moment of my life, I thought I was going to die”

Resolution

- **Definition:** Outcome and consequence of the complicating action
- **Function:** Answers “What finally happened?”
- **Typical position:** Late narrative, after complicating action
- **Linguistic markers:** Outcome verbs (“improved”, “resolved”, “recovered”), result markers (“ended up”, “turned out”)
- **Example:** “The ER gave me glucose and I stabilized within 20 minutes”

Coda

- **Definition:** Return to present, reflection on narrative, or bridge to current situation
- **Function:** Signals narrative closure and connects past to present
- **Typical position:** Final sentence(s)
- **Linguistic markers:** Present tense shift, reflective language (“now I know”, “since then”), future orientation
- **Example:** “Now I always carry glucose tablets with me everywhere I go”

3. BIO Tagging Schema for AAO Extraction

Entity Types and Tags

Agents (who performs or experiences):

- B-AGENT: Beginning of agent entity
- I-AGENT: Inside/continuation of agent entity
- Examples: “I”, “my doctor”, “the nurse”, “my endocrinologist”

Actions (interventions, behaviors, decisions):

- B-ACTION: Beginning of action phrase
- I-ACTION: Inside/continuation of action phrase
- Examples: “started metformin”, “skipped insulin dose”, “went to emergency room”

Outcomes (results, state changes, consequences):

- B-OUTCOME: Beginning of outcome description
- I-OUTCOME: Inside/continuation of outcome description
- Examples: “blood sugar dropped”, “felt better”, “A1C improved to 6.2”

Quantifiers (clinical measurements):

- B-QUANT: Beginning of quantitative measurement
- I-QUANT: Inside/continuation of measurement
- Examples: “2.4 mmol/L”, “140/90 mmHg”, “20 units”

Outside entities:

- O: Token not part of any AAO component

Annotation Examples

Example 1: Simple AAO triplet

Text: “I started metformin and my A1C improved to 6.2”

Token	Tag	Component
**I**	B-AGENT	Agent
**started**	B-ACTION	Action
**metformin**	I-ACTION	Action
**and**	O	-
**my**	O	-
**A1C**	B-OUTCOME	Outcome
**improved**	I-OUTCOME	Outcome
**to**	I-OUTCOME	Outcome
**6.2**	B-QUANT	Quantifier

Triplet: (Agent: “I”, Action: “started metformin”, Outcome: “A1C improved to 6.2”)

Example 2: Outcome-triggers-action (emergency)

Text: “My blood sugar was 2.4 so I rushed to the hospital”

Token	Tag	Component
**My**	O	-
**blood**	B-OUTCOME	Outcome
**sugar**	I-OUTCOME	Outcome
**was**	I-OUTCOME	Outcome
**2.4**	B-QUANT	Quantifier
**so**	O	-
**I**	B-AGENT	Agent
**rushed**	B-ACTION	Action
**to**	I-ACTION	Action
**the**	I-ACTION	Action
**hospital**	I-ACTION	Action

Triplet: (Agent: “I”, Action: “rushed to hospital”, Outcome: “blood sugar was 2.4”)
Causal direction: OUTCOME → TRIGGERS → ACTION (emergency response)

Example 3: Implicit agent (orphan outcome)
Text: “Started herbs. Blood pressure dropped”.

Sentence 1:

Token	Tag	Component
**Started**	B-ACTION	Action
**Herbs**	I-ACTION	Action

Sentence 2:

Token	Tag	Component
**Blood**	B-OUTCOME	Outcome
**Pressure**	I-OUTCOME	Outcome
**Dropped**	I-OUTCOME	Outcome

Processing:
1. Action identified: “Started herbs”
2. Outcome identified: “Blood pressure dropped”
3. No explicit agent → Orphan recovery defaults to narrator
4. Proximity linking (within 50-token window)

Reconstructed Triplet: (Agent: “[narrator]”, Action: “started herbs”, Outcome: “blood pressure dropped”)

4. Annotation Protocol

4.1 Annotator Qualifications
Annotator 1 (Biomedical Expert):
- PhD in Neuroimmunology
- 25 years pharmaceutical research and drug production experience
- Expertise: Medical terminology, drug mechanisms, biochemical processes
- Primary responsibility: Medical accuracy, entity identification, UMLS concept validation

Annotator 2 (Clinical Expert):
- MD with 18 years clinical practice
- Board-certified in General Practitioner and Women’s Health Specialist
- Expertise: Patient communication, clinical plausibility, diagnostic reasoning
- Primary responsibility: Narrative coherence, causal plausibility, clinical context

4.2 Training and Calibration
Phase 1: Guideline Training (2 hours)
- Review theoretical frameworks (Labov, Plutchik, Horne, Street)

- Study operational definitions for all labels
- Review BIO tagging conventions
- Practice with 10 pre-annotated examples with discussion

Phase 2: Calibration (n=100 segments)

- Independent annotation of 100 narrative segments
- Weekly meeting to discuss disagreements
- Iterative guideline refinement based on edge cases
- Target: Achieve $\alpha \geq 0.75$ before proceeding to full annotation

Phase 3: Full Annotation (n=2,000 segments)

- Independent annotation with weekly check-ins
- Flag ambiguous cases for adjudication
- Maintain annotation log documenting decisions

4.3 Two-Stage Annotation Procedure

Stage 1: Narrative and Interpretive Annotation (Sentence-level)

For each sentence in narrative segment:

1. **Labovian Stage:** Assign dominant narrative function
    - If sentence serves multiple functions, select primary function
    - Use context from surrounding sentences
    - Mark as "Mixed" if genuinely ambiguous (for later adjudication)
2. **Emotion Label:** Identify primary emotion expressed
    - Select from: Fear, Hope, Frustration, Relief, Anger (a "None" option was available during annotation but received zero labels; the operational label set therefore comprises the five emotion classes above)
    - If multiple emotions present, select most prominent
    - Consider both explicit emotional words and implicit tone
3. **Stance Label:** Identify narrator's position toward medical recommendations
    - Select from: Compliant, Resistant, Questioning, Advocating, Neutral
    - Focus on behavioral intention, not just stated belief
    - Consider context of full narrative
4. **Tone Label:** Identify communicative register
    - Select from: Clinical, Anxious, Confident, Supportive, Skeptical
    - Consider vocabulary choice, formality, certainty markers
    - May differ from emotion (e.g., anxious tone with hopeful emotion)

Stage 2: AAO Component Extraction (Token-level)

For each token in narrative segment:

1. **Identify Agents:**
    - Who performs actions or experiences outcomes?
    - Include pronouns (I, we, they), names, roles (doctor, nurse)
    - Mark first token as B-AGENT, subsequent as I-AGENT

2. **Identify Actions:**
    - Interventions, behaviors, decisions, treatments
    - Include verb phrase and complements
    - Mark first token as B-ACTION, subsequent as I-ACTION
3. **Identify Outcomes:**
    - Results, state changes, consequences
    - Include condition descriptions and measurements
    - Mark first token as B-OUTCOME, subsequent as I-OUTCOME
4. **Identify Quantifiers:**
    - Clinical measurements (glucose, BP, A1C, dosage)
    - Include value and unit
    - Mark first token as B-QUANT, subsequent as I-QUANT
5. **Link Components:**
    - Maintain chain continuity by noting which Agent→Action→Outcome
    - Use proximity and grammatical cues
    - Flag ambiguous linkages for review

4.4 Edge Case Handling

Ambiguous Agency:

- Default to most plausible actor based on context
- If narrative perspective shifts, mark agent explicitly
- Passive constructions: infer agent from context or mark as "[unspecified]"

Multiple Emotional Content:

- Prioritize primary emotion for mutual exclusivity
- Note secondary emotions in annotation comments
- If truly mixed (e.g., hopeful + anxious), mark as "Mixed" for adjudication

Overlapping Narrative Functions:

- Adopt dominant-function approach for consistency
- Sentences simultaneously serving Evaluation + Resolution → assign to whichever is more prominent
- Document decision rationale in annotation log

Implicit Causal Links:

- Mark action and outcome even if causal connective absent
- Use proximity (within 50 tokens) as default linking criterion
- Trust human judgment about implied causality
- Causal direction analyzer will computationally verify

Non-narrative Content:

- Short responses, acknowledgments, questions without narrative structure: Mark as "Non-narrative"

- These segments contribute any extracted AAO spans to their thread-level graph but are flagged during annotation for adjudication; in the final corpus only 8 of 2,000 segments (0.4%) yielded no AAO span. Graph generation occurs at the thread level, aggregating spans across each thread's segments, so no segment-level exclusion step is applied

4.5 Quality Control

Weekly Adjudication Sessions:

- Review flagged ambiguous cases
- Discuss disagreements and reach consensus
- Update guidelines based on emerging patterns
- Track common error types

Inter-rater Reliability Monitoring:

- Calculate Krippendorff's alpha every 200 annotations
- Target: $\alpha \geq 0.67$ (acceptable), $\alpha \geq 0.75$ (good), $\alpha \geq 0.80$ (excellent)
- If α drops below 0.67, pause for recalibration

Final Adjudication:

- All disagreements resolved through consensus discussion
- Maintain log of adjudication decisions with rationale
- Final dataset: Only consensus labels used for model training

5. Inter-rater Reliability Results

By Dimension

Dimension	Krippendorff's Alpha	Agreement %	Interpretation
**Labovian Stages**	0.81	88.9%	Excellent
**Emotion**	0.79	87.2%	Good
**Stance**	0.78	86.5%	Good
**Tone**	0.78	86.3%	Good
**AAO Agents**	0.82	89.4%	Excellent
**AAO Actions**	0.77	85.9%	Good
**AAO Outcomes**	0.75	84.6%	Good

Overall Assessment: All dimensions exceeded α=0.67 threshold for acceptable reliability [34]. Labovian stages and Agent identification achieved excellent reliability ($\alpha \geq 0.80$), while other dimensions achieved good reliability ($\alpha \geq 0.75$).

Disagreement Analysis

Total Disagreements: 246 of 2,000 segments (12.3%)

By Type:

1. **Narrative stage boundaries** (n=92, 37.4%): Overlapping functions (Evaluation + Resolution)
2. **Emotion ambiguity** (n=68, 27.6%): Mixed emotional content (Hope + Anxiety)
3. **Stance interpretation** (n=51, 20.7%): Questioning vs. Resistant distinction
4. **Agent identification** (n=35, 14.2%): Implicit pronouns, passive constructions

Resolution: All disagreements resolved through consensus adjudication sessions. Decisions documented in annotation log.

6. Annotation Statistics

Coverage

Metric	Value
**Source corpus threads**	5,386
**Annotated threads / narrative segments**	704 / 2,000
**Segments yielding ≥1 AAO span**	1,992 (99.6%)
**Total tokens annotated**	82,826
**Segments with complete AAO triplet (Agent+Action+Outcome)**	1,625 (81.2%)
**Segments yielding zero AAO spans**	8 (0.4%)

Note: The 2,000 annotated segments were drawn from 704 annotated narratives, each corresponding to one thread in the annotation lineage and indexed by comment_id, the grouping key used for cross-validation (a re-indexed integer key; see the main text, Limitations, regarding identifier provenance), sampled from the full corpus of 5,386 threads. AAO extraction operates at the segment level: 1,992 of 2,000 segments (99.6%) yielded at least one AAO span, and 1,625 (81.2%) yielded a complete Agent-Action-Outcome triplet; only 8 segments (0.4%) yielded no AAO span. Narrative graphs are then constructed at the thread level, with each of the 704 threads aggregating the AAO triplets extracted from its constituent segments into a single graph. The figure 704 therefore denotes the number of annotated threads (and resulting narrative graphs), not a count of segments retained after filtering. No two-thirds exclusion of segments occurs; segment-level AAO coverage is near-complete.

Label Distribution

Percentages are rounded half up to one decimal place from the exact proportions and may not sum to exactly 100.0%; counts sum to 2,000 for every dimension.

Labovian Stages (n=2,000 segments):

- Orientation: 852 (42.6%)
- Evaluation: 420 (21.0%)
- Abstract: 331 (16.6%)
- Complicating Action: 146 (7.3%)
- Resolution: 144 (7.2%)
- Coda: 107 (5.4%)

Emotion (n=2,000 segments):

- Fear: 775 (38.8%)
- Frustration: 595 (29.8%)
- Hope: 403 (20.2%)
- Relief: 166 (8.3%)
- Anger: 61 (3.1%)

Stance (n=2,000 segments):

- Neutral: 834 (41.7%)
- Resistant: 551 (27.6%)

- Compliant: 337 (16.9%)
- Advocating: 163 (8.2%)
- Questioning: 115 (5.8%)

Tone (n=2,000 segments):
- Confident: 607 (30.4%)
- Clinical: 581 (29.1%)
- Anxious: 579 (29.0%)
- Supportive: 152 (7.6%)
- Skeptical: 81 (4.1%)

7. References for Theoretical Frameworks

[13] Labov W, Waletzky J. Narrative analysis: oral versions of personal experience. In: Helm J, ed. Essays on the Verbal and Visual Arts. University of Washington Press; 1967:12-44.

[22] Plutchik R. Emotion: A Psychoevolutionary Synthesis. Harper & Row; 1980.

[23] Plutchik R. A general psychoevolutionary theory of emotion. In: Plutchik R, Kellerman H, eds. Emotion: Theory, Research, and Experience. Vol 1. Academic Press; 1980:3-33.

[24] Horne R, Weinman J. Patients' beliefs about prescribed medicines and their role in adherence to treatment in chronic physical illness. J Psychosom Res. 1999;47(6):555-567.

[25] Street RL Jr, Makoul G, Arora NK, Epstein RM. How does communication heal? Pathways linking clinician-patient communication to health outcomes. Patient Educ Couns. 2009;74(3):295-301.

[27] Labov W. The transformation of experience in narrative syntax. In: Language in the Inner City: Studies in the Black English Vernacular. University of Pennsylvania Press; 1972:354-396.

[29] DiMatteo MR. Variations in patients' adherence to medical recommendations: a quantitative review of 50 years of research. Med Care. 2004;42(3):200-209.

[30] Epstein RM, Street RL Jr. Patient-Centered Communication in Cancer Care: Promoting Healing and Reducing Suffering. National Cancer Institute; 2007. NIH Publication No. 07-6225.

[34] Krippendorff K. Content Analysis: An Introduction to Its Methodology. 4th ed. SAGE Publications; 2018.
