# Supplementary material for: AI-Driven Reconstruction of Online Health Narratives Using Agent-Action-Outcome Modeling: Algorithm Development and Validation
Source: JMIR AI. 2026 Nov 13;5:e83496. doi: 10.2196/83496 (PMC13766579; doi:10.2196/83496)
Supplement: Supplementary file 2 [file ai-v5-e83496-s002.pdf]

Multimedia Appendix 2

Model Hyperparameters, Ablation Results, and Statistical Comparisons

1. BioBERT+CRF AAO Extractor Hyperparameters

The BioBERT+CRF model performs BIO sequence tagging to extract Agent, Action, and Outcome spans. A Conditional Random Field (CRF) layer enforces valid BIO transition constraints through a learned transition matrix, replacing per-token argmax decoding with Viterbi decoding.

Table A2.1. BioBERT+CRF Hyperparameters

Parameter	Value
Base model	dmis-lab/biobert-base-cased-v1.2
CRF layer	pytorch-crf (Viterbi decode)
Max sequence length	128 tokens
Batch size	8 (effective 16 with gradient accumulation)
Learning rate	5e-5
Weight decay	0.01
Max epochs	20
Warmup ratio	0.1
Gradient accumulation steps	2
Max gradient norm	1.0
Dropout	0.3
Early stopping patience	7 epochs
Cross-validation	5-fold GroupKFold on comment_id (thread id)
Held-out test size	15% (grouped, reserved before CV)
Random seed	42
First-subword masking	Enabled
BIO tag set	B-AGENT, I-AGENT, B-ACTION, I-ACTION, B-OUTCOME, I-OUTCOME, O

Table A2.2. BioBERT+CRF AAO Extraction Results

Metric	Precision	Recall	F1
**ACTION**	0.947	0.857	0.900
AGENT	0.890	0.787	0.835
OUTCOME	0.790	0.750	0.770
**Micro**	0.892	0.812	0.850
**Macro**	0.876	0.798	0.835

Held-out test set: 286 segments from 100 unseen threads, 2,138 gold entity spans. 5-fold grouped CV span-F1: 0.851 +/- 0.007.

2. DistilBERT Multi-Task Classifier Hyperparameters

The DistilBERT multi-task classifier predicts four interpretive dimensions (Labovian stage, emotion, stance, tone) from shared encoder representations. Task-specific linear heads project the [CLS] embedding to each label space.

Table A2.3. DistilBERT Hyperparameters

Parameter	Value
Base model	distilbert-base-uncased
Max sequence length	256 tokens
Batch size	16
Learning rate	3e-5
Weight decay	0.01
Max epochs	15
Warmup steps	200
Early stopping patience	5 epochs
Task loss weights	labov: 1.0, emotion: 0.8, tone: 0.8, stance: 0.8
Cross-validation	5-fold GroupKFold on comment_id (thread id)
Random seed	42

Table A2.4. DistilBERT Classification Results (5-fold Grouped CV)

Task	Macro-F1	SD	Classes
**Labov stage**	0.501	0.043	6
Emotion	0.466	0.032	5
Tone	0.465	0.023	5
Stance	0.528	0.036	5

Canonical results from the primary 5-fold grouped CV training run of the joint multi-task model. These values derive from a different training run than the matched-fold comparison in Table A2.5 and are therefore not expected to be numerically identical to it. Standard deviations reported with the cross-validation macro-F1 values (and the AAO extraction CV span-F1 of 0.851 +/- 0.007 in the main text and Table A2.2) are population standard deviations across the five folds (ddof=0). The 95% confidence intervals in Table A2.5 are paired-t intervals and use the sample standard deviation (ddof=1) of the per-fold deltas, as the t interval requires.

3. Single-Task vs Multi-Task Comparison

To assess whether multi-task learning provides a performance benefit, we conducted a matched-fold paired comparison using the same encoder, hyperparameters, and grouped folds. The single-task configuration trains a separate model per task; the multi-task configuration trains all four heads jointly with shared encoder weights.

Table A2.5. Multi-Task vs Single-Task DistilBERT (Matched-Fold Comparison)

Task	Multi F1	Single F1	Delta	95% CI (Δ)	Paired t p	Wilcoxon p
**Labov**	0.493	0.516	-0.023	[-0.060, +0.013]	0.155	0.188
Emotion	0.493	0.487	+0.005	[-0.066, +0.077]	0.850	0.812
Tone	0.490	0.507	-0.017	[-0.081, +0.047]	0.504	0.625
Stance	0.549	0.572	-0.024	[-0.057, +0.010]	0.119	0.188

These values derive from a separate multi-task model instance trained specifically for the matched-fold single-versus-multi-task comparison (so that per-fold pairing with the single-task models is valid); they are therefore not expected to match the canonical run reported in Table A2.4. No significant multi-task benefit observed on any task. Single-task achieves higher macro-F1 on 3 of 4 tasks. The 95% CIs are paired-t intervals on the mean per-fold delta (multi minus single, across the five grouped folds) and include zero for every task, confirming that no multi-task benefit is demonstrated. The shared encoder is retained for computational efficiency (one forward pass for four tasks), not for performance gains.

4. Statistical Model Comparison (McNemar’s Test)

Three AAO extraction systems were compared on the same grouped held-out test set (286 segments, 100 unseen threads, 2,138 gold entity spans). McNemar’s test with continuity correction replaces paired t-tests to avoid fold-overlap independence violations.

Table A2.6. Pairwise McNemar's Test Results

Comparison	n01	n10	Statistic	p-value
TF-IDF vs BioBERT (no CRF)	41	398	288.69	< 0.001
TF-IDF vs BioBERT + CRF	41	410	300.27	< 0.001
BioBERT (no CRF) vs BioBERT + CRF	43	55	1.23	0.266

n01 = spans correct by first system only; n10 = spans correct by second system only. The CRF significantly outperforms TF-IDF (P < .001) but provides a non-significant improvement over BioBERT without CRF (P = .266). For reference, absolute span-level F1 on this held-out test set (286 segments, 100 threads), computed for all three systems under the strict seqeval span convention consistent with the McNemar analysis, was 0.781 for TF-IDF + Logistic Regression, 0.841 for BioBERT without CRF, and 0.850 for BioBERT + CRF. Gold-span counts differ by system because tokenization differs (2,267 gold spans for the TF-IDF pipeline; 2,138 for the two transformer systems), so each F1 is computed against its own system's gold spans.

5. Heuristic Component Validation

Error analyses were conducted on three rule-based pipeline components using hand-labeled stratified samples.

Table A2.7. Orphan Recovery Validation

Metric	Value	95% CI
Precision (n=50)	0.640	[0.500, 0.780]
Estimated recall (40 narratives scanned)	~0.97	3 missed entities

Table A2.8. Clinical-Value Regex Validation

Metric	Value	95% CI
Precision (n=120)	0.900	[0.842, 0.950]
Recall (48 true values in 50 narratives)	0.604	[0.458, 0.729]

Table A2.9. Causal Direction Validation (n=143 hand-labeled segments)

Class	P	R	F1	tp/fp/fn
action_causes_outcome	0.575	0.426	0.489	23/17/31
outcome_triggers_action	0.277	0.361	0.313	13/34/23
bidirectional	0.000	0.000	-	0/27/5
unknown	0.586	0.354	0.442	17/12/31

Overall accuracy: 0.371. Inversion (OTA) precision 95% CI: [0.149, 0.404]. The causal direction distribution is reported as a descriptive characteristic from an exploratory heuristic. A learned causal direction model is identified as future work.

6. UMLS Normalization Statistics

The hybrid UMLS normalizer achieved 100% concept coverage across 12,101 normalization queries through a three-stage cascade:

Stage	Count	Percentage
SQLite exact matching	7,769	64.2%
SQLite fuzzy matching (threshold >= 0.7)	3,185	26.3%
BioBERT semantic matching (cosine >= 0.6)	1,147	9.5%
Unmatched	0	0.0%

BioBERT semantic matches: mean cosine similarity = 0.9499, SD = 0.0182. The tight distribution indicates high-confidence semantic resolution.

7. Domain-Stratified AAO Extraction Performance

AAO span extraction on the grouped held-out test set (286 segments from 100 unseen threads, 2,138 gold entity spans) stratified by the health-topic domain assigned by the pipeline topic model. Span-level precision, recall, and F1 (seqeval, micro) are reported per domain. Subreddit membership was not propagated to the segment-level annotation file, so stratification uses the pipeline-assigned topic; per-subreddit stratification is identified as future work.

Domain (topic model)	Test segments	Test threads	Gold spans	Precision	Recall	Span-F1
**Diabetes**	125	43	981	0.888	0.817	0.851
**Hypertension**	90	33	676	0.893	0.806	0.848
**General health**	71	24	481	0.899	0.811	0.852
**Overall**	286	100	2,138	0.892	0.812	0.850

Extraction performance is stable across domains (per-domain span-F1 0.848-0.852), with no domain differing from another by more than 0.004; the micro-pooled overall span-F1 across all domains is 0.850.

Per-domain segment and thread counts sum to the held-out totals (286 segments, 100 threads, 2,138 gold spans).
