# Supplementary material for: AI-Driven Reconstruction of Online Health Narratives Using Agent-Action-Outcome Modeling: Algorithm Development and Validation
Source: JMIR AI. 2026 Nov 13;5:e83496. doi: 10.2196/83496 (PMC13766579; doi:10.2196/83496)
Supplement: Supplementary file 3 [file ai-v5-e83496-s003.pdf]

Multimedia Appendix 3: Production Implementations of Causal Direction Detection, Clinical Quantifier Extraction, and Orphan Recovery

The functions below are reproduced verbatim from the deposited production pipeline, aao_pipeline_production_v3_4.py, the file that generated every result reported in the main text and in Multimedia Appendix 2. Line ranges refer to that file so that each block can be checked against the deposited source. No pseudocode, abstraction, or idealization is presented: what follows is the code that ran.

The causal direction detector returns one of four categories (action causes outcome, outcome triggers action, bidirectional, unknown), and the four-category distribution reported in the main text and in Table A2.9 is the output of this function. Clinical severity thresholds, as encoded in CLINICAL_THRESHOLDS, are based on the American Diabetes Association Standards of Care in Diabetes 2024 for glucose and HbA1c, with severe hypoglycemia set at <2.8 mmol/L (50 mg/dL), below the ADA level 2 threshold of 3.0 mmol/L (54 mg/dL), and on the 2017 ACC/AHA hypertension guideline for blood pressure. The entries of the module-level CONFIG dictionary (lines 84-86 and 91) that govern linking distances and the default agent are:

```
CONFIG = {
    # ... (other pipeline settings omitted)
    "MAX_LINKING_DISTANCE": 50,
    "USE_NEAREST_NEIGHBOR_FALLBACK": True,
    "DEFAULT_AGENT_FOR_ORPHANS": "narrator",
    "LINK_QUANTIFIERS_TO_OUTCOMES": True,
    # ...
}
```

Linking uses MAX_LINKING_DISTANCE (50 tokens) for agent-to-action and action-to-outcome links, MAX_LINKING_DISTANCE // 2 (25 tokens) for quantifier-to-outcome and instrument or temporal attachment, and MAX_LINKING_DISTANCE * 2 (100 tokens) for orphan-outcome recovery, in either direction.

1. Causal Direction Detection (lines 140-268)

```
class CausalDirection(Enum):
    ACTION_CAUSES_OUTCOME = "action_causes_outcome"
    OUTCOME_TRIGGERS_ACTION = "outcome_triggers_action"
    BIDIRECTIONAL = "bidirectional"
    UNKNOWN = "unknown"

class CausalDirectionAnalyzer:
    """Detects causal direction in health narratives via linguistic markers,
    temporal ordering, emergency indicators, and clinical severity."""

    OUTCOME_TRIGGERS_MARKERS = [
        "had to", "have to", "needed to", "must", "forced to",
        "made me", "caused me to", "so i", "so we", "therefore",
        "thus", "as a result", "consequently", "went to", "rushed to",
        "called", "visited", "was taken to", "was rushed to", "was admitted",
    ]

    ACTION_CAUSES_MARKERS = [
```

```
    "resulted in", "led to", "caused", "produced", "gave me",
    "made me feel", "helped with", "improved", "worsened",
    "increased", "decreased", "reduced", "lowered", "raised",
    "now i", "after taking", "since starting", "effect was",
    "side effect",
]

EMERGENCY_PATTERNS = [
    r"rushed to (the )?(hospital|er|emergency|icu)",
    r"called (911|ambulance|doctor)",
    r"was (hospitalized|admitted|taken to)",
]

CRITICAL_VALUE_PATTERNS = [
    (r"blood sugar.*?(\d+\.?\d*)\s*(mmol|mg)", "blood_sugar"),
    (r"(\d+\.?\d*)\s*(mmol/l|mg/dl)", "glucose_value"),
    (r"(\d+)/(\d+)\s*(mmhg)?", "blood_pressure"),
    (r"a1c.*?(\d+\.?\d*)\s*%?", "a1c"),
]

def __init__(self):
    self.emergency_regexes = [
        re.compile(p, re.IGNORECASE) for p in self.EMERGENCY_PATTERNS
    ]
    self.value_regexes = [
        (re.compile(p, re.IGNORECASE), name)
        for p, name in self.CRITICAL_VALUE_PATTERNS
    ]

def analyze(
    self, text: str, entities: Dict, tokens: List[str]
) -> Dict[str, Any]:
    text_lower = text.lower()
    analysis = {
        "direction": CausalDirection.UNKNOWN,
        "confidence": 0.0,
        "evidence": [],
        "markers_found": [],
        "temporal_order": None,
        "is_emergency_response": False,
        "critical_values": [],
    }

    outcomes = entities.get("outcomes", [])
    actions = entities.get("actions", [])
    if not outcomes or not actions:
        return analysis

    # Emergency patterns
    for regex in self.emergency_regexes:
        if regex.search(text_lower):
            analysis["is_emergency_response"] = True
            analysis["evidence"].append("Emergency pattern detected")
            break

    # Critical values
    for regex, value_type in self.value_regexes:
        match = regex.search(text_lower)
        if match:
            analysis["critical_values"].append(
                {"type": value_type, "value": match.group(0),
                 "position": match.start()}
            )
```

```
        # Score markers
        ot_score, ac_score = 0, 0
        for marker in self.OUTCOME_TRIGGERS_MARKERS:
            if marker in text_lower:
                ot_score += 1
                analysis["markers_found"].append(("outcome_triggers", marker))
        for marker in self.ACTION_CAUSES_MARKERS:
            if marker in text_lower:
                ac_score += 1
                analysis["markers_found"].append(("action_causes", marker))

        # Temporal order
        first_outcome = min(o["start_pos"] for o in outcomes)
        first_action = min(a["start_pos"] for a in actions)
        if first_outcome < first_action:
            analysis["temporal_order"] = "outcome_first"
            ot_score += 2
        else:
            analysis["temporal_order"] = "action_first"
            ac_score += 1

        # Emergency + critical value = strong inverted signal
        if analysis["is_emergency_response"] and analysis["critical_values"]:
            ot_score += 3
            analysis["evidence"].append(
                "Critical value followed by emergency response"
            )

        # Determine direction
        total = ot_score + ac_score
        if total == 0:
            analysis["direction"] = CausalDirection.UNKNOWN
            analysis["confidence"] = 0.0
        elif ot_score > ac_score:
            analysis["direction"] = CausalDirection.OUTCOME_TRIGGERS_ACTION
            analysis["confidence"] = ot_score / (total + 1)
        elif ac_score > ot_score:
            analysis["direction"] = CausalDirection.ACTION_CAUSES_OUTCOME
            analysis["confidence"] = ac_score / (total + 1)
        else:
            analysis["direction"] = CausalDirection.BIDIRECTIONAL
            analysis["confidence"] = 0.5

        return analysis
```

2. Clinical Quantifier Extraction and Classification (lines 275-524)

```
QUANTIFIER_PATTERNS = [
    (r"\b(\d+\.?\d*)\s*(mmol/l|mmol)\b", "glucose_mmol"),
    (r"\b(\d+\.?\d*)\s*(mg/dl)\b", "glucose_mg"),
    (r"\b(\d{2,3})/(\d{2,3})\s*(mmhg)?\b", "blood_pressure"),
    (r"\b(a1c|hba1c).*?(\d+\.?\d*)\s*%?\b", "a1c"),
    (r"\b(\d+\.?\d*)\s*(kg|lbs?|pounds?)\b", "weight"),
    (r"\b(\d+)\s*(bpm)\b", "heart_rate"),
    (r"\b(\d+\.?\d*)\s*(mg|mcg|ml|units?)\b", "dosage"),
    (r"\b(once|twice|one time|two times?|three times?|\d+\s*times?)\b", "frequency"),
]

COMPILED_QUANTIFIER_PATTERNS = [
    (re.compile(p, re.IGNORECASE), name) for p, name in QUANTIFIER_PATTERNS
]

# ADA Standards of Care in Diabetes, 2024 / AHA blood pressure guidelines
```

```python
CLINICAL_THRESHOLDS = {
    "glucose_mmol": {
        "hypoglycemia_severe": 2.8, "hypoglycemia": 3.9,
        "normal_high": 5.5, "hyperglycemia": 11.1,
    },
    "glucose_mg": {
        "hypoglycemia_severe": 50, "hypoglycemia": 70,
        "normal_high": 100, "prediabetes": 126, "hyperglycemia": 200,
    },
    "a1c": {
        "normal": 5.7, "prediabetes": 6.4,
        "diabetes": 6.5, "uncontrolled": 9.0,
    },
    "blood_pressure": {
        "normal": (120, 80), "elevated": (130, 80),
        "stage1": (140, 90), "stage2": (180, 120),
    },
}


def classify_clinical_value(value, value_type: str) -> Dict[str, Any]:
    """Classify a clinical value against evidence-based thresholds.

    Sources:
        Glucose / A1C: ADA Standards of Care in Diabetes, 2024.
        Blood pressure: AHA/ACC Hypertension Guidelines, 2017.
    """
    classification = {
        "value": value, "type": value_type,
        "category": "unknown", "risk_level": "unknown",
        "clinical_significance": None,
    }

    if value_type == "glucose_mmol" and isinstance(value, (int, float)):
        t = CLINICAL_THRESHOLDS["glucose_mmol"]
        if value < t["hypoglycemia_severe"]:
            classification.update({
                "category": "severe_hypoglycemia", "risk_level": "critical",
                "clinical_significance":
                    "Life-threatening low blood sugar requiring immediate intervention",
            })
        elif value < t["hypoglycemia"]:
            classification.update({
                "category": "hypoglycemia", "risk_level": "high",
                "clinical_significance":
                    "Low blood sugar requiring treatment",
            })
        elif value <= t["normal_high"]:
            classification.update({"category": "normal", "risk_level": "low"})
        elif value > t["hyperglycemia"]:
            classification.update({
                "category": "hyperglycemia", "risk_level": "high",
                "clinical_significance":
                    "High blood sugar requiring attention",
            })
        else:
            classification.update({
                "category": "above_normal", "risk_level": "moderate",
            })

    elif value_type == "glucose_mg" and isinstance(value, (int, float)):
        t = CLINICAL_THRESHOLDS["glucose_mg"]
        if value < t["hypoglycemia_severe"]:
            classification.update({
                "category": "severe_hypoglycemia", "risk_level": "critical",
                "clinical_significance":
                    "Life-threatening low blood sugar requiring immediate intervention",
```

```
            })
        elif value < t["hypoglycemia"]:
            classification.update({
                "category": "hypoglycemia", "risk_level": "high",
                "clinical_significance":
                    "Low blood sugar requiring treatment",
            })
        elif value <= t["normal_high"]:
            classification.update({"category": "normal", "risk_level": "low"})
        elif value <= t["prediabetes"]:
            classification.update({
                "category": "prediabetes_range", "risk_level": "moderate",
                "clinical_significance":
                    "Fasting glucose in prediabetes range",
            })
        elif value > t["hyperglycemia"]:
            classification.update({
                "category": "hyperglycemia", "risk_level": "high",
                "clinical_significance":
                    "High blood sugar requiring attention",
            })
        else:
            classification.update({
                "category": "diabetes_range", "risk_level": "high",
                "clinical_significance":
                    "Fasting glucose in diabetes range",
            })

    elif value_type == "a1c" and isinstance(value, (int, float)):
        t = CLINICAL_THRESHOLDS["a1c"]
        if value < t["normal"]:
            classification.update({"category": "normal", "risk_level": "low"})
        elif value < t["diabetes"]:
            classification.update({
                "category": "prediabetes", "risk_level": "moderate",
                "clinical_significance": "A1C in prediabetes range",
            })
        elif value < t["uncontrolled"]:
            classification.update({
                "category": "diabetes", "risk_level": "high",
                "clinical_significance": "A1C indicates diabetes",
            })
        else:
            classification.update({
                "category": "uncontrolled_diabetes", "risk_level": "critical",
                "clinical_significance":
                    "A1C indicates uncontrolled diabetes requiring urgent review",
            })

    elif value_type == "blood_pressure" and isinstance(value, tuple) and len(value) ==
2:
        systolic, diastolic = value
        bp = CLINICAL_THRESHOLDS["blood_pressure"]
        if systolic < bp["normal"][0] and diastolic < bp["normal"][1]:
            classification.update({"category": "normal", "risk_level": "low"})
        elif systolic < bp["elevated"][0] and diastolic < bp["elevated"][1]:
            classification.update({
                "category": "elevated", "risk_level": "moderate",
                "clinical_significance": "Elevated blood pressure",
            })
        elif systolic < bp["stage1"][0] and diastolic < bp["stage1"][1]:
            classification.update({
                "category": "stage1_hypertension", "risk_level": "high",
                "clinical_significance":
                    "Stage 1 hypertension requiring lifestyle modification",
            })
```

```python
        elif systolic >= bp["stage2"][0] or diastolic >= bp["stage2"][1]:
            classification.update({
                "category": "hypertensive_crisis", "risk_level": "critical",
                "clinical_significance":
                    "Hypertensive crisis requiring immediate medical attention",
            })
        else:
            classification.update({
                "category": "stage2_hypertension", "risk_level": "high",
                "clinical_significance":
                    "Stage 2 hypertension requiring medication",
            })

    return classification

def extract_quantifiers_from_text(
    text: str, tokens: List[str]
) -> List[Dict]:
    """Extract clinical quantifiers from narrative text."""
    quantifiers = []
    text_lower = text.lower()

    for pattern, value_type in COMPILED_QUANTIFIER_PATTERNS:
        for match in pattern.finditer(text_lower):
            matched_text = match.group(0)
            numeric_value = None

            try:
                if value_type == "blood_pressure":
                    groups = match.groups()
                    if len(groups) >= 2:
                        numeric_value = (float(groups[0]), float(groups[1]))
                else:
                    for group in match.groups():
                        if group and re.match(r"^\d+\.?\d*$", str(group)):
                            numeric_value = float(group)
                            break
            except (ValueError, TypeError):
                pass

            start_char = match.start()
            token_pos = find_token_position_for_char(text, tokens, start_char)

            quantifier = {
                "text": matched_text.strip(),
                "type": value_type,
                "numeric_value": numeric_value,
                "start_pos": token_pos,
                "end_pos": token_pos + len(matched_text.split()) - 1,
                "char_start": start_char,
                "char_end": match.end(),
            }

            if numeric_value and value_type in [
                "glucose_mmol", "glucose_mg", "a1c", "blood_pressure"
            ]:
                classify_type = value_type
                quantifier["clinical_classification"] = classify_clinical_value(
                    numeric_value, classify_type
                )

            quantifiers.append(quantifier)

    return remove_overlapping_quantifiers(quantifiers)
```

```
def find_token_position_for_char(
    text: str, tokens: List[str], char_pos: int
) -> int:
    current_char = 0
    for i, token in enumerate(tokens):
        token_clean = token.replace("##", "")
        if current_char <= char_pos < current_char + len(token_clean) + 1:
            return i
        current_char += len(token_clean) + 1
    return len(tokens) - 1


def remove_overlapping_quantifiers(quantifiers: List[Dict]) -> List[Dict]:
    if not quantifiers:
        return []

    def _priority(q):
        has_class = 1 if "clinical_classification" in q else 0
        return (q["char_start"], -has_class, -len(q["text"]))

    sorted_q = sorted(quantifiers, key=_priority)
    result = []
    occupied = []
    for q in sorted_q:
        s, e = q["char_start"], q["char_end"]
        if not any(not (e <= os or s >= oe) for os, oe in occupied):
            result.append(q)
            occupied.append((s, e))
    return sorted(result, key=lambda x: x["char_start"])
```

3. Entity Linking and Orphan Recovery (lines 792-958)

find_nearest_entity implements the directional distance search used throughout triplet assembly. extract_aao_triplets_v34 assembles agent-driven triplets, links quantifiers to outcomes, and, in its final block, recovers outcomes left unlinked by assigning the default agent and the nearest action within the recovery window.

```
def find_nearest_entity(
    target_pos: int, entity_list: List[Dict],
    used_indices: Set, direction: str = "forward",
    max_distance: int = None,
) -> Tuple[Optional[Dict], Optional[int]]:
    max_distance = max_distance or CONFIG.get("MAX_LINKING_DISTANCE", 50)
    best_entity, best_idx, best_dist = None, None, float("inf")

    for idx, entity in enumerate(entity_list):
        if idx in used_indices:
            continue
        if direction == "forward":
            dist = entity["start_pos"] - target_pos
            if dist < 0:
                continue
        elif direction == "backward":
            dist = target_pos - entity["end_pos"]
            if dist < 0:
                continue
        else:  # both
            dist = min(
                abs(entity["start_pos"] - target_pos),
                abs(entity["end_pos"] - target_pos),
            )
```

```python
        if dist < best_dist and dist <= max_distance:
            best_entity, best_idx, best_dist = entity, idx, dist
    return best_entity, best_idx

def extract_aao_triplets_v34(
    entities: Dict, causal_analysis: Dict, tokens: List[str]
) -> List[Dict]:
    """Assemble AAO triplets with causal direction awareness."""
    triplets = []
    agents = entities.get("agents", [])
    actions = entities.get("actions", [])
    outcomes = entities.get("outcomes", [])
    instruments = entities.get("instruments", [])
    temporals = entities.get("temporals", [])
    quantifiers = entities.get("quantifiers", [])

    used_actions: Set[int] = set()
    used_outcomes: Set[int] = set()
    used_instruments: Set[int] = set()
    used_temporals: Set[int] = set()
    max_dist = CONFIG.get("MAX_LINKING_DISTANCE", 50)
    causal_dir = causal_analysis.get("direction", CausalDirection.UNKNOWN)
    invert = causal_dir == CausalDirection.OUTCOME_TRIGGERS_ACTION

    # Link quantifiers to nearest outcomes
    quantifier_outcome_map: Dict[int, Dict] = {}
    if CONFIG.get("LINK_QUANTIFIERS_TO_OUTCOMES", True):
        for _, quantifier in enumerate(quantifiers):
            best_out, out_idx = find_nearest_entity(
                quantifier["start_pos"], outcomes, set(), "both", max_dist // 2
            )
            if best_out and out_idx is not None:
                quantifier_outcome_map[out_idx] = quantifier

    # Agent-driven triplet assembly
    for agent in agents:
        best_action, act_idx = find_nearest_entity(
            agent["end_pos"], actions, used_actions, "forward", max_dist
        )
        if best_action and act_idx is not None:
            used_actions.add(act_idx)

        search_pos = best_action["end_pos"] if best_action else agent["end_pos"]
        if invert:
            best_out, out_idx = find_nearest_entity(
                agent["start_pos"], outcomes, used_outcomes, "backward", max_dist
            )
            if not best_out:
                best_out, out_idx = find_nearest_entity(
                    search_pos, outcomes, used_outcomes, "forward", max_dist
                )
        else:
            best_out, out_idx = find_nearest_entity(
                search_pos, outcomes, used_outcomes, "forward", max_dist
            )

        if best_out and out_idx is not None:
            used_outcomes.add(out_idx)

        if best_action or best_out:
            triplet = {
                "agent": agent["text"], "agent_pos": agent["start_pos"],
                "causal_direction": causal_dir.value,
            }
            if best_action:
```

```
                triplet.update({
                    "action": best_action["text"],
                    "action_pos": best_action["start_pos"],
                })
                # Attach instrument/temporal near the action
                inst, inst_idx = find_nearest_entity(
                    best_action["start_pos"], instruments,
                    used_instruments, "both", max_dist // 2,
                )
                if inst and inst_idx is not None:
                    used_instruments.add(inst_idx)
                    triplet.update({
                        "instrument": inst["text"],
                        "instrument_pos": inst["start_pos"],
                    })
                temp, temp_idx = find_nearest_entity(
                    best_action["start_pos"], temporals,
                    used_temporals, "both", max_dist // 2,
                )
                if temp and temp_idx is not None:
                    used_temporals.add(temp_idx)
                    triplet.update({
                        "temporal": temp["text"],
                        "temporal_pos": temp["start_pos"],
                    })
            if best_out:
                triplet.update({
                    "outcome": best_out["text"],
                    "outcome_pos": best_out["start_pos"],
                })
                if out_idx in quantifier_outcome_map:
                    q = quantifier_outcome_map[out_idx]
                    triplet["quantifier"] = q["text"]
                    triplet["quantifier_pos"] = q["start_pos"]
                    if "clinical_classification" in q:
                        triplet["clinical_classification"] =
q["clinical_classification"]
            triplets.append(triplet)

    # Orphan recovery: unlinked outcomes
    if CONFIG.get("USE_NEAREST_NEIGHBOR_FALLBACK", True):
        for k, outcome in enumerate(outcomes):
            if k in used_outcomes:
                continue
            nearest_action, min_dist = None, float("inf")
            for action in actions:
                dist = abs(outcome["start_pos"] - action["end_pos"])
                if dist < min_dist:
                    min_dist, nearest_action = dist, action

            triplet = {
                "outcome": outcome["text"],
                "outcome_pos": outcome["start_pos"],
                "orphan_recovered": True,
                "causal_direction": causal_dir.value,
            }
            if CONFIG.get("DEFAULT_AGENT_FOR_ORPHANS"):
                triplet.update({
                    "agent": CONFIG["DEFAULT_AGENT_FOR_ORPHANS"],
                    "agent_pos": -1,
                })
            if nearest_action and min_dist <= max_dist * 2:
                triplet.update({
                    "action": nearest_action["text"],
                    "action_pos": nearest_action["start_pos"],
                    "action_distance": min_dist,
```

```
            })
        if k in quantifier_outcome_map:
            q = quantifier_outcome_map[k]
            triplet["quantifier"] = q["text"]
            if "clinical_classification" in q:
                triplet["clinical_classification"] = q["clinical_classification"]
        triplets.append(triplet)
        used_outcomes.add(k)

    return triplets
```
