# Supplementary material for: AI-Driven Reconstruction of Online Health Narratives Using Agent-Action-Outcome Modeling: Algorithm Development and Validation
Source: JMIR AI. 2026 Nov 13;5:e83496. doi: 10.2196/83496 (PMC13766579; doi:10.2196/83496)
Supplement: Supplementary file 4 [file ai-v5-e83496-s004.pdf]

Multimedia Appendix 4: Expert Validation Rubric and Independent Single-Rater Assessments

1. Expert Validation Methodology

1.1 Sample Selection

Sample Size: n=100 narrative graphs

Stratification:

- By pipeline-assigned topic domain: proportional to corpus distribution across the three domains produced by the pipeline topic model (diabetes 46.4%, hypertension 31.1%, general health 22.4%; computed from the pipeline topic assignments over all 704 annotated threads), consistent with the domain-stratified performance reported in Multimedia Appendix 2, Section 7. Obesity- and COVID-19-related discussions are present in the collected corpus (obesity and ivermectin/COVID-19 were among the keyword search targets used during collection). The pipeline topic model applied to the 704-narrative annotated subset produced three domains (diabetes, hypertension, and general health), with no standalone obesity or COVID-19 cluster; the stratification below therefore reflects that three-domain output rather than the collection keywords.
- By complexity: equal representation of simple (1-2 triplets), moderate (3-5 triplets), and complex (6+ triplets) narratives.
- By causal direction: representation across the four categories reported in the main text at the segment level (action-to-outcome 51.8%, outcome-to-action 17.3%, bidirectional 7.8%, undetermined 23.2%). These are segment-level proportions (N=2,000 segments); because sampling was performed at the thread level (100 of 704 graphs), the causal-direction stratification is approximate.

Selection Method: stratified random sampling from the 704 complete narrative graphs generated by the pipeline.

1.2 Expert Rater Credentials

Rater 1: Biomedical Research Expert

- Credentials: PhD in Neuroimmunology
- Experience: 25 years pharmaceutical research and drug production
- Expertise domains: medical terminology, drug mechanisms, biochemical processes, UMLS concept accuracy
- Primary evaluation role: narrative coherence assessment

Rater 2: Clinical Practice Expert

- Credentials: MD, Board-Certified General Practitioner; Specialist in Obstetrics and Gynaecology
- Experience: 18 years clinical practice
- Expertise domains: patient communication, clinical plausibility, diagnostic reasoning, treatment protocols
- Primary evaluation role: medical plausibility assessment

1.3 Evaluation Procedure

Materials Provided to Raters:

1. Original narrative text: full Reddit post/comment as it appears to readers
2. Generated AAO graph: JSON representation with nodes (agents, actions, outcomes, quantifiers) and edges (PERFORMS, RESULTS_IN, TRIGGERS, HAS_VALUE)
3. Metadata: UMLS CUIs, confidence scores, causal direction labels, interpretive dimensions (emotion, stance, tone)
4. Rating instructions: this rubric with anchored examples

Rating Process:

- Independent evaluation: each rater evaluates all 100 graphs independently without consultation. Rater 1 assessed narrative coherence only; Rater 2 assessed medical plausibility only.
- Anchored scale: two dimensions (narrative coherence, medical plausibility) on 0-1 continuous scales with anchored descriptions at 0.0, 0.5, and 1.0
- No forced distribution: raters may assign any score
- Time per graph: average 4-6 minutes per graph evaluation

2. Rating Scales with Anchored Descriptions

2.1 Narrative Coherence Scale (Rater 1, Biomedical Research Expert)

Definition: the degree to which the generated AAO graph preserves the logical flow, causal relationships, and narrative structure of the original health story as a human reader would understand it.

Score 0.0 - Completely Incoherent: the graph fundamentally misrepresents the narrative. Causal relationships are backwards, agents are misidentified, or the story logic is broken beyond recognition (e.g., an emergency response labeled as planned treatment; a critical causal inversion in which going to hospital is represented as causing hypoglycemia rather than responding to it).

Score 0.5 - Partially Coherent: the graph captures some narrative elements correctly but contains significant errors or omissions that distort meaning. A human could partially reconstruct the story but key elements are wrong or missing (e.g., core causal structure correct, but missing quantifier extraction and an emotion misclassification).

Score 1.0 - Fully Coherent: the graph accurately and completely represents the narrative's logical flow, causal relationships, and structural elements. All agents, actions, and outcomes are correctly identified with appropriate boundaries; causal direction is correct; quantifiers are linked; interpretive dimensions align with text; and temporal relationships are preserved.

2.2 Medical Plausibility Scale (Rater 2, Clinical Practice Expert)

Definition: the degree to which the extracted entities, relationships, and medical concepts align with clinical knowledge, treatment protocols, and medical plausibility.

Score 0.0 - Medically Implausible: the graph contains medically impossible relationships or fundamental clinical errors that would mislead automated credibility systems (e.g., a UMLS mapping error assigning insulin to a hypertension concept, or severity misclassification labeling target-range glucose as hypoglycemia).

Score 0.5 - Partially Plausible: the graph represents clinically reasonable relationships with minor errors or ambiguities that do not fundamentally compromise medical validity (e.g., correct medication class but wrong specific drug; a missing but non-contradictory causal link).

Score 1.0 - Fully Plausible: the graph accurately represents clinically valid relationships, treatment protocols, and medical concepts. UMLS mappings are precise, quantifier severity assessments align with clinical guidelines, and causal direction matches medical reasoning.

3. Independent Single-Rater Score Distributions

Because the two raters evaluated different constructs (Rater 1: narrative coherence; Rater 2: medical plausibility), a between-rater agreement statistic is not defined: coherence and plausibility are distinct dimensions, not repeated measurements of one quantity. We therefore report each rater's assessment independently and do not compute Krippendorff's alpha, Pearson, or Spearman correlations across the two dimensions. (The annotator inter-rater reliability for the shared annotation labels is reported separately in Multimedia Appendix 1, Section 5.)

3.1 Score Distribution by Dimension

Score Range	Narrative Coherence (n=100)	Medical Plausibility (n=100)
Mean	0.89	0.91
Median	0.92	0.94
Std Dev	0.08	0.07

Key findings:

- Narrative coherence (Rater 1): mean 0.89, median 0.92, SD 0.08, n=100.
- Medical plausibility (Rater 2): mean 0.91, median 0.94, SD 0.07, n=100.
- The nine lowest-scoring graphs on either dimension are analysed in Section 3.3.

3.2 Cases of Largest Divergence Between the Two Independent Scores

The table below lists the graphs for which the coherence and plausibility scores diverged most. Because the two scores measure different constructs, this divergence is descriptive only and is not interpreted as rater disagreement.

Graph ID	Coherence (R1)	Plausibility (R2)	|difference|	Note
Graph 23	0.6	0.9	0.3	R1 penalized a missing temporal marker; R2 focused on the correct medical relationships
Graph 47	0.5	0.8	0.3	Ambiguous causal direction (the connective 'so'); R1 read it as an error, R2 as acceptable in context
Graph 61	0.7	0.95	0.25	Emotion classification ('anxious hope'); R1 penalized forced single-label, R2 focused on medical content

The largest coherence-plausibility differences observed were 0.30 (Graphs 23 and 47) and 0.25 (Graph 61); no graph exceeded 0.30.

3.3 Qualitative Failure Mode Analysis (Low-Scoring Graphs, n=9)

The nine lowest-scoring graphs, analysed for failure mode.

Graph ID	Coherence	Plausibility	Primary Failure Mode	Example Issue
Graph 12	0.65	0.88	Incorrect causal direction	'My blood pressure dropped after I took the medication' misidentified as outcome-to-action instead of action-to-outcome due to temporal marker confusion
Graph 23	0.60	0.90	Missing temporal marker	Symptoms prompting a clinic visit were extracted, but the temporal connective linking the two events was not represented, weakening sequence reconstruction; medical relationships were preserved
Graph 34	0.68	0.85	Missed implicit agent	'Started metformin. Felt better'. Failed to link narrator as agent across sentence boundary
Graph 47	0.50	0.80	Ambiguous causality	'I was dizzy so I checked my blood sugar and it was low' - unclear whether dizziness caused checking or low glucose caused dizziness
Graph 55	0.70	0.72	UMLS mapping imprecision	'Blood pressure medicine' mapped to generic antihypertensive class instead of the specific drug mentioned earlier
Graph 61	0.70	0.95	Emotion misclassification	'I'm cautiously optimistic' labeled as Hope instead of recognizing mixed Hope+Fear
Graph 73	0.68	0.90	Missing quantifier link	A1C value extracted but not linked to appropriate outcome node
Graph 82	0.65	0.88	Incomplete entity extraction	Compound phrase 'emergency room blood glucose test' partially extracted
Graph 91	0.67	0.70	Multi-agent confusion	Doctor-patient dialogue with unclear speaker attribution for one action

Patterns:

- Most common failure (3/9): causal or temporal representation errors, comprising incorrect causal direction, a missing temporal marker, and genuinely ambiguous causality.
- Agent attribution failures (2/9): implicit agent recovery across sentence boundaries and speaker attribution in multi-agent dialogue.
- Remaining failures (4/9): an unlinked quantifier, a partially extracted compound phrase, UMLS mapping imprecision, and forced single-label emotion classification.
- Medical plausibility remained generally higher: even graphs with structural errors preserved medically valid relationships (mean plausibility 0.84 vs. coherence 0.65 for low-scoring cases).

4. Expert Qualitative Feedback

4.1 Comments on High-Quality Graphs (Score >= 0.90)

Rater 1 (Biomedical Expert): "This graph excellently captures the emergency nature of the hypoglycemic episode. The outcome-to-action causal direction is correctly identified, quantifiers are precisely linked, and the emotional arc from fear to relief is accurately represented."

Rater 2 (Clinical Expert): "Medically comprehensive. All treatment recommendations align with current guidelines. The graph would enable accurate credibility assessment because the quantified glucose values are correctly mapped to clinical severity thresholds."

4.2 Comments on Low-Quality Graphs (Score <= 0.70)

Rater 1 on Graph 47 (0.50 coherence): "The narrative contains genuinely ambiguous causality: 'I was dizzy so I checked my blood sugar and it was low.' Did dizziness cause the checking, or did low blood sugar cause dizziness which then prompted checking? The graph forced a choice, but human readers would recognize this as ambiguous. This suggests a need for uncertainty representation."

Rater 2 on Graph 91 (0.70 plausibility): "The medical relationships are accurate but incomplete. The graph misses that the doctor's prescription was motivated by the patient's reported symptoms. This causal link is clinically important for assessing appropriateness of treatment."

5. Validation Conclusions

5.1 Overall Assessment

The expert review indicates that the Narrative Builder pipeline produces high-quality narrative graphs suitable for downstream credibility assessment:

- Narrative coherence (Rater 1): mean 0.89 (excellent range).
- Medical plausibility (Rater 2): mean 0.91 (excellent range).
- Success rate: the large majority of graphs scored >= 0.70 on both dimensions; the nine lowest-scoring graphs are analysed in Section 3.3.
- Clinical validity: medical plausibility scores were consistently high, indicating reliable medical concept extraction and relationship modeling.

5.2 Identified Limitations

The review also reveals specific failure modes for future improvement:

1. Causal or temporal representation errors (3 cases): incorrect causal direction, a missing temporal marker, and genuinely ambiguous causality.
2. Agent attribution (2 cases): pronoun omission across sentence boundaries and speaker attribution in doctor-patient exchanges.
3. Extraction and mapping gaps (3 cases): an unlinked quantifier, a partially extracted compound phrase, and UMLS mapping imprecision.
4. Mixed emotional states (1 case): forced single-label classification for genuinely mixed emotions.

These represent 9% of validated graphs, indicating mature pipeline performance with clear improvement targets.
